# CRISPR/Cas9-Mediated Mutagenesis of Four Putative Symbiosis Genes of the Tropical Tree *Parasponia andersonii* Reveals Novel Phenotypes

**DOI:** 10.3389/fpls.2018.00284

**Published:** 2018-03-06

**Authors:** Arjan van Zeijl, Titis A. K. Wardhani, Maryam Seifi Kalhor, Luuk Rutten, Fengjiao Bu, Marijke Hartog, Sidney Linders, Elena E. Fedorova, Ton Bisseling, Wouter Kohlen, Rene Geurts

**Affiliations:** Laboratory of Molecular Biology, Department of Plant Sciences, Wageningen University & Research, Wageningen, Netherlands

**Keywords:** *Parasponia andersonii*, rhizobium, nodule, symbiosis, CRISPR/Cas9, stable transformation

## Abstract

*Parasponia* represents five fast-growing tropical tree species in the Cannabaceae and is the only plant lineage besides legumes that can establish nitrogen-fixing nodules with rhizobium. Comparative analyses between legumes and *Parasponia* allows identification of conserved genetic networks controlling this symbiosis. However, such studies are hampered due to the absence of powerful reverse genetic tools for *Parasponia*. Here, we present a fast and efficient protocol for *Agrobacterium tumefaciens*-mediated transformation and CRISPR/Cas9 mutagenesis of *Parasponia andersonii*. Using this protocol, knockout mutants are obtained within 3 months. Due to efficient micro-propagation, bi-allelic mutants can be studied in the T_0_ generation, allowing phenotypic evaluation within 6 months after transformation. We mutated four genes – *PanHK4, PanEIN2, PanNSP1*, and *PanNSP2* – that control cytokinin, ethylene, or strigolactone hormonal networks and that in legumes commit essential symbiotic functions. Knockout mutants in *Panhk4* and *Panein2* displayed developmental phenotypes, namely reduced procambium activity in *Panhk4* and disturbed sex differentiation in *Panein2* mutants. The symbiotic phenotypes of *Panhk4* and *Panein2* mutant lines differ from those in legumes. In contrast, *PanNSP1* and *PanNSP2* are essential for nodule formation, a phenotype similar as reported for legumes. This indicates a conserved role for these GRAS-type transcriptional regulators in rhizobium symbiosis, illustrating the value of *Parasponia* trees as a research model for reverse genetic studies.

## Introduction

*Parasponia* are tropical tree species belonging to the Cannabis family (Cannabaceae) and are known as the only non-legume plants that can establish a nitrogen-fixing endosymbiosis with rhizobium (Clason, 1936; Trinick, 1973; Akkermans et al., 1978). The *Parasponia* genus consists of five species indigenous to the Malay Archipelago and Papua New Guinea, where they grow on the slopes of volcanic mountains (Clason, 1936; Soepadmo, 1974; Becking, 1992). *Parasponia* spp. are typical fast-growing pioneer plants, capable of covering nitrogen-poor eroded soils in a relatively short time span (Becking, 1992). Under suitable greenhouse conditions, young *Parasponia* trees can grow at speeds exceeding 45 centimeters per month, and fix up to 850 kg N ha^-1^ year^-1^ in association with rhizobium (Trinick, 1980, 1981; Trinick and Hadobas, 1989). As *Parasponia* is the only non-legume that can establish rhizobium symbiosis, it may represent a valuable model to study the core genetic networks underlying this symbiosis (Geurts et al., 2012, 2016; Behm et al., 2014).

Like legumes, *Parasponia* develops specialized root nodular organs to host the rhizobium partner. Nodules provide the rhizobium bacteria with suitable environmental conditions to convert atmospheric nitrogen into ammonium. The Cannabaceae and legume family (Fabaceae) diverged about a 100 million years ago (Wang et al., 2009), underlining that the rhizobium symbiosis in legumes and *Parasponia* evolved largely independent (Li et al., 2015). This is reflected in the distinct nodule-types found in both lineages (Behm et al., 2014). Legume nodules possess a large central zone of infected cells, which is surrounded by peripheral vascular bundles. In contrast, *Parasponia* nodules have a central vascular bundle and infected cells in the peripheral zone, giving these nodules a lateral root-like appearance. Nevertheless, initial comparative studies revealed that both symbioses are founded on conserved signaling networks. In legumes as well as *Parasponia*, root nodule formation is induced upon recognition of rhizobial secreted lipo-chitooligosaccharide (LCO) signals (Marvel et al., 1987; Op den Camp et al., 2011; Granqvist et al., 2015). Research on model legumes, like *Medicago truncatula* and *Lotus japonicus*, showed that the perception of these symbiotic signals requires a signaling cascade that has been co-opted from the much older endomycorrhizal symbiosis (Geurts et al., 2012; Oldroyd, 2013). In legumes, activation of the LCO signaling network results in a massive transcriptional reprogramming, requiring among others the GRAS-type transcriptional regulators NODULATION SIGNALLING PATHWAY 1 (NSP1) and NSP2 and the cytokinin receptor MtCRE1/LjLHK1 (Kaló et al., 2005; Smit et al., 2005; Gonzalez-Rizzo et al., 2006; Heckmann et al., 2006; Murray et al., 2007; Tirichine et al., 2007; Plet et al., 2011). Subsequent nodule formation is tightly controlled by regulatory feedback loops, including negative regulation by ethylene signaling (Penmetsa et al., 2008; Miyata et al., 2013; van Zeijl et al., 2015b).

A reference quality genome sequence for *Parasponia andersonii* and draft genome sequences of two additional *Parasponia* species have been generated (van Velzen et al., 2017). Mining these genomes uncovered ∼1,800 putative symbiosis genes, of which 100s are close homologs of legume symbiosis genes (van Velzen et al., 2017). Initial reverse genetic studies in *P. andersonii*, using a transient *Agrobacterium rhizogenes*-based root transformation system, revealed that at least two genes – *NOD FACTOR PERCEPTION 1* (*PanNFP1*) and *CALCIUM AND CALMODULIN-DEPENDENT PROTEIN KINASE* (*PanCCaMK*) – commit conserved functions in the *Parasponia* and legume LCO signaling pathways (Op den Camp et al., 2011). We argue that a more comprehensive comparative analysis between legumes and *Parasponia* will allow identification of conserved genetic networks that are essential to establish symbiosis with rhizobium. However, to use *Parasponia* as an effective research model – alongside the legume models *M. truncatula* and *L. japonicus* – efficient transformation and genome editing tools are required.

Here, we exploit an efficient *in vitro* micro-propagation system available for *P. andersonii* to establish stable transformation and CRISPR/Cas9-mediated mutagenesis for this species (Davey et al., 1993; Webster et al., 1995; Cao et al., 2012). We show that using *Agrobacterium tumefaciens*-mediated transformation, stable transgenic lines of *P. andersonii* can be obtained in ∼3–4 months. Additionally, we show that *P. andersonii* is amenable to targeted mutagenesis using the CRISPR/Cas9 system. As ∼40% of the resulting T_0_ lines harbor bi-allelic mutations, these can be phenotyped upon *in vitro* propagation. As proof of concept, we mutated four genes in *P. andersonii* that in legumes control hormonal pathways as well as commit symbiotic functions. These include: the GRAS-type transcriptional regulators *NSP1* and *NSP2* that are essential for nodule organogenesis (Kaló et al., 2005; Smit et al., 2005; Heckmann et al., 2006) and control strigolactone biosynthesis by mediating *DWARF27* (*D27*) expression (Liu et al., 2011; van Zeijl et al., 2015a); the cytokinin receptor *HISTIDINE KINASE 4* (*HK4*) that in legumes is essential for nodule organogenesis (Gonzalez-Rizzo et al., 2006; Murray et al., 2007; Plet et al., 2011); and the ethylene signaling hub *ETHYLENE INSENSITIVE 2* (*EIN2*) that is a negative regulator of nodulation in legumes (Penmetsa and Cook, 1997; Penmetsa et al., 2008; Miyata et al., 2013).

## Materials and Methods

### Plant Materials and Growth Conditions

All experiments were conducted using *P. andersonii* WU1 or offspring thereof (Op den Camp et al., 2011; van Velzen et al., 2017). *P. andersonii* trees were grown in a conditioned greenhouse at 28°C, 85% humidity and a 16/8 h day/night regime. For *in vitro* culturing, *P. andersonii* was grown in an Elbanton growth cabinet at 28°C, 16/8 h day/night. Growth of young *P. andersonii* plantlets for nodulation assays or qRT-PCR analysis was performed in 1 L crystal-clear polypropelene containers equipped with a gas exchange filter (OS140BOX, Duchefa Biochemie, Netherlands). Pots were half-filled with agraperlite (Maasmond-Westland, Netherlands) and watered with modified EKM medium [3 mM MES (C_6_H_13_NO_4_) pH 6.6, 2.08 mM MgSO_4_, 0.88 mM KH_2_PO_4_, 2.07 mM K_2_HPO_4_, 1.45 mM CaCl_2_, 0.70 mM Na_2_SO_4_, 0.375 mM NH_4_NO_3_, 15 μM Fe-citrate, 6.6 μM MnSO_4_, 1.5 μM ZnSO_4_, 1.6 μM CuSO_4_, 4 μM H_3_BO_3_, 4.1 μM Na_2_MoO_4_] (Becking, 1983) and placed in a climate room set at 28°C, 16/8 h day/night. For nodulation assays, EKM medium was inoculated with *Mesorhizobium plurifarium* BOR2 (OD_600_ = 0.025) (van Velzen et al., 2017).

### Vectors and Constructs

For CRISPR/Cas9-mediated mutagenesis, binary transformation constructs were created using Golden Gate assembly (Engler et al., 2009). For an overview of all Golden Gate clones used in this study, see Supplementary Table 1. sgRNAs were designed based on the principles described in Doench et al. (2014) and PCR amplified using specific forward primers and a universal reverse primer (Supplementary Table 2), using Addgene plasmid # 46966 as template (Nekrasov et al., 2013). These were cloned behind the AtU6p small RNA promoter and inserted behind the neomycin phosphotransferease II gene (*NPTII*) and an *Arabidopsis thaliana* codon-optimized variant of Cas9 (Fauser et al., 2014) fused to an N-terminal nuclear localization signal and driven by the 35S promoter (Supplementary Table 1). As negative control, a binary vector was created containing only the NPTII- and NLS-Cas9-encoding sequences (Supplementary Table 1). To setup *P. andersonii* stable transformation, vector pKGWFS7-RR was used (Karimi et al., 2002).

### Phylogenetic Reconstruction

Protein sequences of *Glycine max* (Wm82.a2.v1) (Schmutz et al., 2010), *M. truncatula* (Mt4.0v1) (Young et al., 2011; Tang et al., 2014) and *Populus trichocarpa* (v3.0) (Tuskan et al., 2006) were obtained through Phytozome 10^1^. Protein sequences of *P. andersonii* (PanWU01x14_asm01_ann01) and *Trema orientalis* (TorRG33x02_asm01_ann01) were obtained from www.parasponia.org (van Velzen et al., 2017). These sequences were mined using sequences from *A. thaliana* (TAIR10^2^) (Lamesch et al., 2012) and *M. truncatula*. Protein sequences were aligned using MAFFT v7.017 (Katoh et al., 2002) implemented in Geneious 8.1.9 (Biomatters, New Zealand), using default parameter settings. Approximately-maximum-likelihood phylogenetic trees were constructed using FastTree (Price et al., 2009) implemented in Geneious 8.1.9. Mid-point rooting was applied for better tree visualization using FigTree v1.4.2^3^.

### Plant Transformation

Stable transformation of *P. andersonii* was performed using *A. tumefaciens* strain AGL1 (Lazo et al., 1991). *A. tumefaciens* was grown for 2 days on agar-solidified LB medium containing appropriate antibiotics. For each *P. andersonii* transformation, two Petri dishes (Ø 9 cm) of *A. tumefaciens* were used. Bacteria were scraped from plate and resuspended in 25 ml of infiltration medium [SH10 (Supplementary Table 3), 20 mg/l acetosyringone (Sigma, United States), 0.001% (v/v) Silwet L-77^4^]. *P. andersonii* tissue explants used for transformation were harvested from mature trees grown under greenhouse conditions and sterilized in 2% commercial bleach for 15 min. Tissue explants were cut at both ends inside the *A. tumefaciens* suspension, creating fresh wound surfaces, and kept inside the suspension for about 20 min. Subsequently, excess liquid was removed from tissue explants using sterilized filter paper and explants were placed on co-cultivation medium [Root-inducing medium (Supplementary Table 3), 20 mg/l acetosyringone (Sigma, United States)]. Plates were incubated for 2 days at 21°C in darkness. After 2 days, tissue explants were washed three times using SH10 (Supplementary Table 3) and subsequently dried using filter paper. Tissue explants were placed on root-inducing medium containing 50 mg/l kanamycin and 300 mg/l cefotaxime and incubated at 28°C, 16/8 h day/night. Nine days after transformation, tissue explants were transferred to propagation medium (Supplementary Table 3) containing 50 mg/l kanamycin and 300 mg/l cefotaxime. Plates were refreshed every other week. When regenerative calli reached ∼2 mm in size they were separated from tissue explants to stimulate shoot formation. A single shoot was selected per tissue explant. These shoots were propagated on propagation medium (Supplementary Table 3), as previously described (Cao et al., 2012). Rooted plantlets were generated by placing individual shoots on root-inducing medium (Supplementary Table 3) (Cao et al., 2012).

### Characterization of Transgenic Lines

For T-DNA copy number estimates based on qPCR analysis, genomic DNA was isolated using the DNeasy Plant Mini Kit (Qiagen, Germany). qPCR was set up in a 10 μl reaction system with 2x iQ SYBR Green Super-mix (Bio-Rad, United States) and 5 ng template DNA. The experimental setup and procedure were executed on a CFX Connect optical cycler, according to the manufacturer’s protocol (Bio-Rad, United States). T-DNA copy number was estimated using two primer pairs amplifying part of the T-DNA and two primer pairs amplifying single copy *P. andersonii* genes (*PanAGT1* and PanWU01x14_asm01_ann01_338920) that were selected based on a study by Duarte et al. (2010). Primer sequences are listed in Supplementary Table 2. Data analysis was performed using CFX Manager 3.0 software (Bio-Rad, United States). For T-DNA copy number estimates based on Southern blotting, genomic DNA was separately digested with *XbaI, HindIII*, and *EcoRI*. Blots were hybridized with a 516 bp α-32P-labeled probe corresponding to part of the *NPTII* gene that was amplified using primers nptII_Fw and nptII_Rv listed in Supplementary Table 2.

Genotyping of transgenic lines was performed using the Phire Plant Direct PCR Kit (Thermo Scientific, United States) and gene specific primers listed in Supplementary Table 2. Ploidy estimates of transgenic lines were determined by FACS as described by van Velzen et al. (2017).

To determine ethylene sensitivity of *Panein2* mutants, tips of young branches of 4 months-old trees were covered with 1 L plastic bags and injected with 1 ml of pure ethylene gas. After 3 days, bags were removed and leaf abscission examined. Total number of leaves on treated branches varied from 6 to 18.

### Microtome Sectioning

Stem cross-sections were made from the primary stem, 5 cm below the apical meristem, of 2 month-old trees. Shoot tissue was fixed in 5% glutaraldehyde and embedded in Technovit 7100 (Heraeus-Kulzer, Germany), according to the manufacturer’s protocol. Semi-thin (7 μm) sections were cut using a microtome (Reichert-Jung, Leica Microsystems, Netherlands) and stained with 0.05% Toluidine Blue O. Images were taken using a Leica DM5500B microscope equipped with a DFC425C camera (Leica Microsystems, Germany). Average procambium cell number was quantified by averaging the number of cells within 25–40 cell files for each of the biological replicates.

Nodule tissue fixation and embedding was performed as previously described (Fedorova et al., 1999). Semi-thin (0.6 μm) sections were cut using a Leica Ultracut microtome (Leica Microsystems, Germany) and photographed as described above.

### RNA Isolation and qRT-PCR Analysis

RNA was isolated from snap-frozen root tips (∼2–3 cm) as described by van Velzen et al. (2017). cDNA was prepared from 1 μg of total RNA using the i-script cDNA synthesis kit (Bio-Rad, United States), following the manufacturer’s instructions. RT-qPCR was set up as described above. Normalization was performed based on two stably expressed reference genes [*UNKNOWN 2* (*PanUNK2*) and *ELONGATION FACTOR 1α* (*PanEF1α*)], chosen based on previous study (Czechowski et al., 2005; Bansal et al., 2015). All primer sequences are listed in Supplementary Table 2.

### Statistical Analysis

Statistical differences were determined based on one-way ANOVA and Tukey *post hoc* tests. Statistical analyses were performed using IBM SPSS Statistics 23.0 (IBM, United States).

## Results

### *Agrobacterium tumefaciens*-Mediated Transformation of *Parasponia*

To establish a protocol for stable transformation of *P. andersonii*, we first determined the most optimal conditions for regeneration of non-transgenic tissue. We compared regeneration efficiencies of nine tissue explant types in combination with 11 different media, including the propagation and root-inducing media previously used for *P. andersonii* (Supplementary Tables 4, 5) (Op den Camp et al., 2011; Cao et al., 2012). This revealed that young stem pieces and petioles placed on original propagation medium regenerate plantlets most efficiently (Supplementary Table 4). Next, we questioned whether stem pieces and petioles could be transformed efficiently using *A. tumefaciens*. To this end, we used *A. tumefaciens* AGL1 carrying a binary transformation vector containing in its T-DNA the kanamycin resistance gene *NPTII* and the red fluorescent protein *DsRED1*. Co-cultivation of *A. tumefaciens* and *P. andersonii* stem or petiole explants was conducted in darkness for 2 days at 21°C to promote T-DNA transfer (Cao et al., 2012). Afterward, tissue explants were placed on selective medium and incubated at 28°C in the light. These latter conditions are most favorable for *P. andersonii* regeneration (Cao et al., 2012). From day 8 onwards, DsRED1-fluorescent cells could be observed near the wound surface indicating a successful transfer of the T-DNA.

**FIGURE 1:**
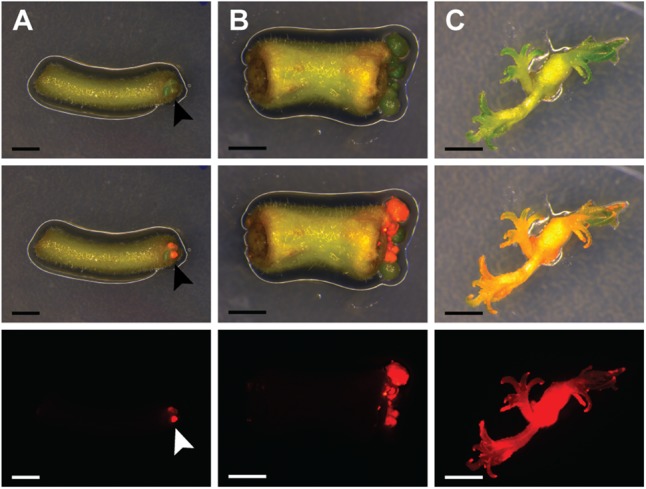
*Parasponia andersonii* stem and petiole explants can be efficiently transformed using *Agrobacterium tumefaciens*. **(A)** Petiole explant at 4 weeks after transformation using *A. tumefaciens*. Arrowheads indicate transgenic micro-calli. **(B)** Stem explant at 5 weeks after transformation using *A. tumefaciens*. **(C)** Small transgenic shoots at 10 weeks after transformation. Explants were incubated on root-inducing medium for 9 days, prior to transfer to propagation medium. DsRED fluorescence indicates transgenic tissue. Scale bars are equal to 2.5 mm. Shown from top to bottom are bright-field images, overlays of bright-field and DsRED fluorescence and DsRED fluorescence images.

Recent research on *A. thaliana* showed that acquisition of pluripotency requires activation of a root developmental program (Kareem et al., 2015). We tested whether an initial culturing period on root-inducing medium further improves the transformation efficiency of *P. andersonii*. This showed to be the case (Supplementary Table 6). About half of the explants formed regenerative calli at 4 weeks after co-cultivation (**Figure 1A**). When 2 mm in size, transgenic calli were separated from tissue explants, which stimulated shoot formation (**Figures 1B,C**). Two to three months after the start of transformation, a single shoot was selected from each explant to ensure that the transgenic lines represent independent transformation events. These shoots can be genotyped and vegetatively propagated (Supplementary Figure 1). The latter allows clonal multiplication of individual transgenic lines in a period of ∼4–6 weeks, which means that phenotyping assays could be initiated at ∼4 months after the start of transformation.

To characterize the resulting transgenic *P. andersonii* lines at the molecular level, we selected – based on red fluorescence – 20 independent transformants for further analyses. PCR reactions using primers amplifying a sequence near the right T-DNA border indicated complete T-DNA integration in 19 out of 20 lines (Supplementary Table 7). To determine whether the transformation procedure might affect ploidy level of the regenerated transgenic lines, we estimated genome size based on flow cytometry. This showed no effect of the transformation procedure on the genome size of transgenic lines (Supplementary Table 7). To estimate the number of T-DNA integrations, we used quantitative RT-PCR (qRT-PCR) as well as Southern blotting. This showed an overall low T-DNA copy number, varying between one and three integrations per line (Supplementary Table 7). We selected three transgenic lines with a single T-DNA integration to examine T-DNA stability. In greenhouse-grown trees as well as *in vitro* propagated material, DsRED1 fluorescence could still be observed at 6–12 months after transgenic lines were selected (Supplementary Figures 1, 2). This indicates that *trans*-genes remain stably integrated into the *P. andersonii* genome and actively transcribed, even after multiple rounds of vegetative propagation. Taken together, the protocol described above allows generating *A. tumefaciens*-transformed *P. andersonii* plantlets within 3 months, which can be phenotyped upon vegetative propagation.

### *Parasponia* Is Amenable to CRISPR/Cas9-Mediated Mutagenesis

To test whether CRISPR/Cas9 could be used for targeted mutagenesis in *P. andersonii*, we aimed at mutating the *P. andersonii* putative orthologs of *EIN2, MtCRE1*/*LjLHK1, NSP1*, and *NSP2*. These genes were selected, because they control legume root nodule formation as well as commit essential non-symbiotic functions in hormone homeostasis. Putative orthologs of all four genes were previously identified from the *P. andersonii* genome and named *PanEIN2, PanHK4, PanNSP1*, and *PanNSP2*, respectively (van Velzen et al., 2017). Phylogenetic reconstruction based on protein sequences confirmed that these represent the most likely orthologs of legume symbiotic genes (Supplementary Figures 3–6). To mutate *PanEIN2, PanHK4, PanNSP1* and *PanNSP2*, three single guide RNAs (sgRNAs) targeting *PanHK4* and *PanNSP2* and single sgRNAs targeting *PanEIN2* and *PanNSP1* were placed under an *A. thaliana AtU6* small RNA promoter (Nekrasov et al., 2013). These were cloned into a binary transformation vector containing the *NPTII* kanamycin resistance gene as well as a Cas9-encoding sequence fused to an N-terminal nuclear-localization signal and driven by the CaMV 35S promoter (Engler et al., 2014; Fauser et al., 2014). The resulting constructs as well a control construct containing only the NPTII- and Cas9-encoding sequences were transformed to *P. andersonii* using the method described above. For all constructs, transgenic shoots were obtained, although in case of the construct targeting *PanHK4* regeneration took considerably longer (up to 6 months). Genotyping of regenerated shoots showed that >85% contained the *Cas9* gene, indicating successful transformation. Potential mutations at any of the target sites were identified through PCR amplification and subsequent sequencing of the PCR product. This revealed mutations at the target site in about half of the transgenic shoots examined, of which the majority were bi-allelic (**Table 1**). Most mutations represent small insertions and deletions but also larger deletions and inversions were identified, some of which occur in between two target sites (Supplementary Figures 7–10). In case of *PanHK4*, most mutants contained small in-frame deletions of 3 or 6 bp that most likely do not disrupt protein function. In fact, only two bi-allelic knockout mutants could be identified (Supplementary Figure 8). For the remaining three constructs, multiple bi-allelic knockout mutants were identified of which three individuals were selected for further studies (for an overview of mutant alleles see Supplementary Figures 7–10).

**Table 1:** Mutation frequency in CRISPR/Cas9 transgenic lines.

Target gene	No. of sgRNA’s	No. of lines	Non-mutated^a^	Mutated		
				Bi-allelic	Heterozygous	Unknown^b^
NSP1	1	29	15 (51.7%)	11 (37.9%)	0	3 (10.3%)
NSP2	3	29	13 (44.8%)	10 (34.5%)	3 (10.3%)	3 (10.3%)
EIN2	1	9	1 (11.1%)	6 (66.7%)	2 (22.2%)	0
HK4	3	26	13 (50.0%)	12 (46.2%)	1 (3.8%)	0
**Total**		**93**	**42 (45.2%)**	**39 (41.9%)**	**6 (6.5%)**	**6 (%)**

*^*a*^This includes an unknown number of individuals that are not transgenic. ^*b*^Sequencing of the PCR product indicates that plants are mutated, but the exact mutation and zygosity were not determined.*

For phenotypic evaluation, *P. andersonii* T_0_ transgenic lines are propagated vegetatively. Therefore, we first evaluated whether any of the mutant lines might be chimeric. To this end, tissue samples were taken from at least three different positions and genotyped for the corresponding target mutation. For each of the mutant lines, except *Pannsp2–9*, the same mutations were retrieved, suggesting that genome editing occurred soon after T-DNA integration. In case of *Pannsp2–9*, chimeric mutations were detected at the first of three target sites (Supplementary Figure 10C). However, the nature of the mutations at the second and third target site prevent that gene function could be restored in this line. Therefore, all 11 mutants are suitable for phenotypic evaluation. This proves that CRISPR/Cas9 can be used to efficiently mutagenize *P. andersonii* in the T_0_ generation.

### Non-symbiotic Phenotypes in *Parasponia ein2, hk4, nsp1*, and *nsp2* Mutant Lines

**FIGURE 2:**
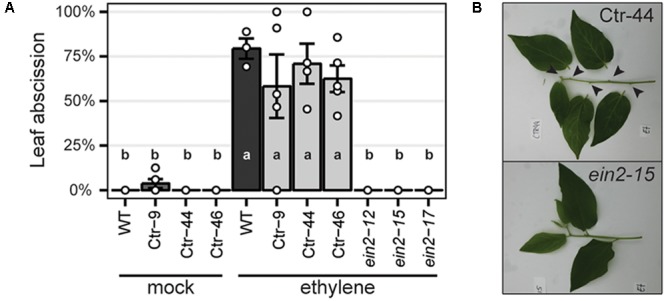
*Panein2* mutants are insensitive to ethylene treatment. **(A)** Percentage of abscised leaves after mock or ethylene treatment. Data represent means of 3–5 biological replicates ± SEM. Dots represent measurement values of biological repeats. Different letters indicate statistical significance (*p* < 0.05) as determined by ANOVA in combination with Tukey *post hoc* test. **(B)** Representative images showing abscission of leaves on a transgenic control line (Ctr-44), but not on a *Panein2* mutant. Abscission points are indicated by arrowheads.

To characterize the resulting *Panein2, Panhk4, Pannsp1* and *Pannsp2* mutant lines, we studied their non-symbiotic phenotypes. *PanEIN2* putatively encodes a central component of the ethylene signaling pathway and therefore *Panein2* mutants are expected to be ethylene insensitive. One phenotype triggered in response to ethylene treatment is abscission of leaves and flowers, as shown in amongst others common bean (*Phaseolus vulgaris*), cotton (*Gossypium hirsutum*), and citrus (*Citrus clementina*) (Jackson and Osborne, 1970; Brown, 1997; Agustí et al., 2009). We exploited this phenotype to assess ethylene sensitivity of *Panein2* mutants. To this end, the tips of young shoot branches of greenhouse grown trees were exposed to ethylene gas. Within 3 days, ethylene triggered abscission of ∼65% of treated leaves on wild-type (WT) *P. andersonii* as well as control transgenic lines (**Figure 2**). In contrast, leaf abscission was not observed on *Panein2* mutant trees (**Figure 2**). This demonstrates that *Panein2* mutants are indeed ethylene insensitive.

**FIGURE 3:**
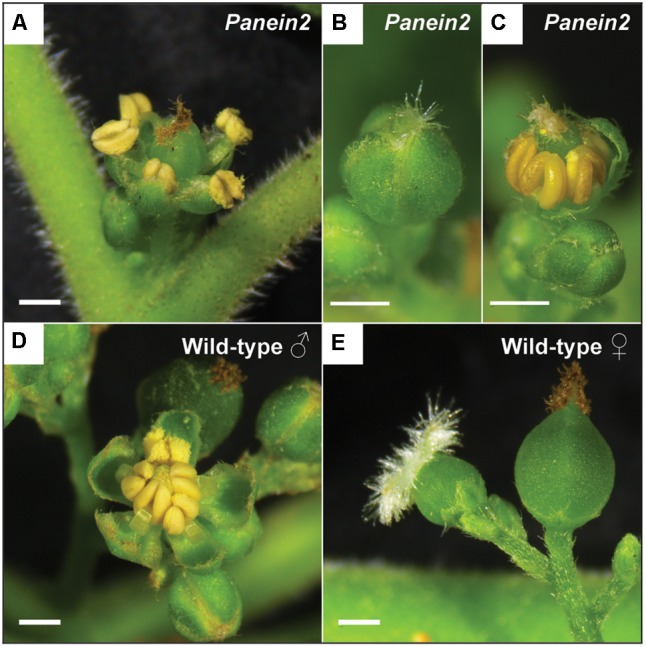
*Panein2* mutants form bisexual flowers. **(A)** Mature *Panein2* flower. Note the presence of both stamen and a carpel inside *Panein2* flowers. **(B)** Immature *Panein2* flower. Note the presence of stigmata, indicating presence of a carpel inside the flower. **(C)** Immature *Panein2* flower of which sepals have been removed to show the presence of stamen. **(D)** Mature wild-type (WT) male flower. **(E)** Mature WT female flowers. Left: young female flower. Right: older female flower. Scale bars are equal to 1 mm.

Inspection of *Panein2* mutant trees revealed an additional non-symbiotic phenotype. These trees form bisexual flowers containing both male and female reproductive organs (**Figures 3A–C**). In contrast, WT *P. andersonii* trees form unisexual flowers that contain either stamens or carpels (Becking, 1992) (**Figures 3D,E**). This suggests that ethylene is involved in the regulation of *Parasponia* sex type.

**FIGURE 4:**
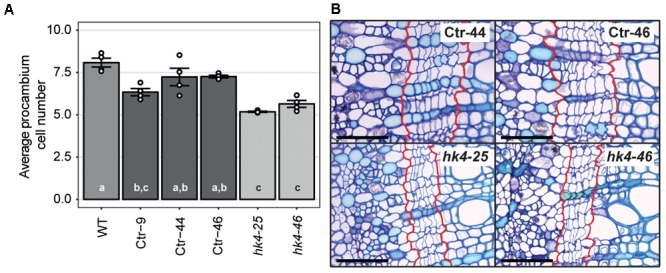
*Panhk4* mutants display reduced procambium activity. **(A)** Average number of procambium cells in WT, transgenic control (Ctr) and *Panhk4* mutant stems. Data represent means of four biological replicates ± SEM. Dots represent measurement values of biological repeats. Different letters indicate statistical significance (*p* < 0.05) as determined by ANOVA in combination with Tukey *post hoc* test. **(B)** Representative images of stem cross-sections of control transgenic (Ctr) and *Panhk4* mutant lines. The location of the procambium is indicated by red lines. Scale bars represent 50 μm.

Cytokinins are important regulators of cambial activity, as shown in *A. thaliana* and poplar (*Populus tremula* x *tremuloides*) (Matsumoto-Kitano et al., 2008; Nieminen et al., 2008; Bhalerao and Fischer, 2017). To determine whether reduced cytokinin sensitivity in *Panhk4* mutant lines affects the activity of the procambium, we sectioned young primary stems, 5 cm below the apical meristem. This showed that procambium activity is reduced in *Panhk4* mutant lines compared to transgenic controls (**Figure 4**). Therefore, we conclude that PanHK4-mediated cytokinin signaling is required for regulation of *P. andersonii* secondary growth.

**FIGURE 5:**
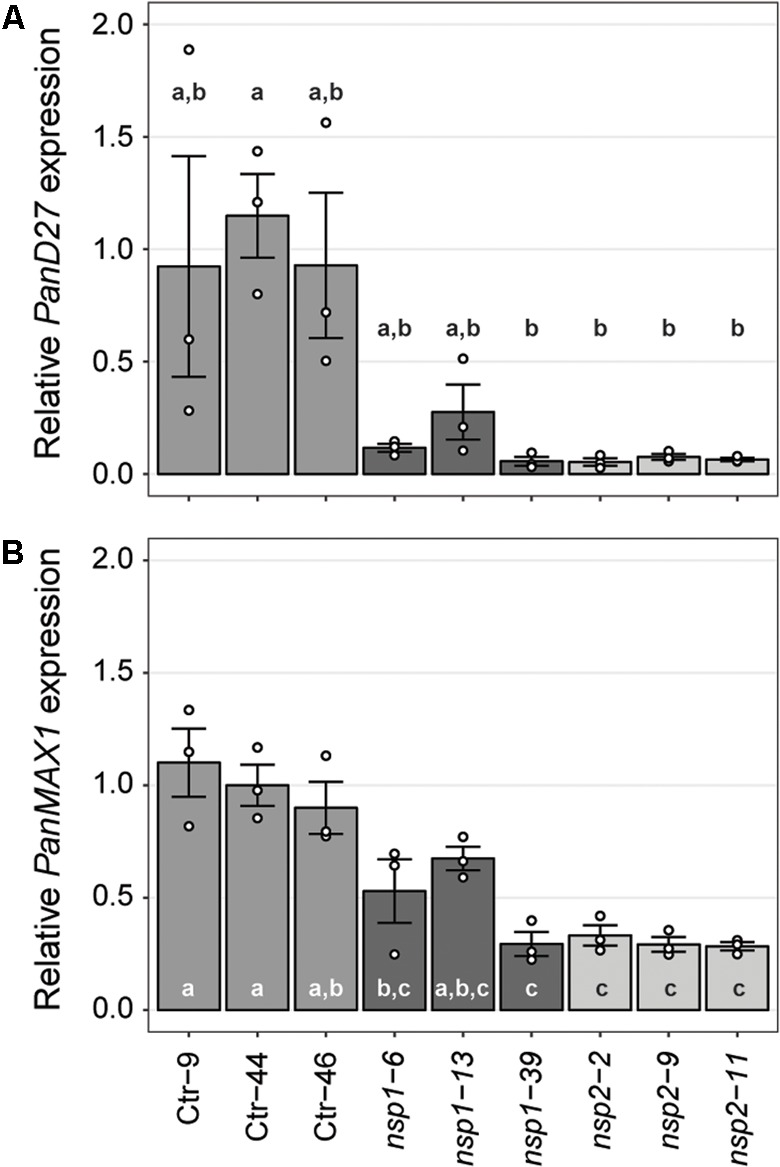
Expression of *PanD27* and *PanMAX1* is reduced in *Pannsp1* and *Pannsp2* mutant roots. Relative expression of *PanD27* **(A)** and *PanMAX1* **(B)** in roots of transgenic control (Ctr) and *Pannsp1* and *Pannsp2* mutant lines. Data represent means of three biological replicates ± SEM. Dots represent measurement values of biological repeats. Different letters indicate statistical significance (*p* < 0.05) as determined by ANOVA in combination with Tukey *post hoc* test.

Expression studies in *M. truncatula* previously identified a set of genes downregulated in roots of *Mtnsp1* and *Mtnsp2* mutants (Liu et al., 2011). Among these are *DWARF27* (*MtD27*; Medtr1g471050) and *MORE AXILLARY BRANCHING 1* (*MtMAX1*; Medtr3g104560) that are putatively involved in strigolactone biosynthesis (Liu et al., 2011; Cardoso et al., 2014; Zhang et al., 2014; van Zeijl et al., 2015a). We identified putative *P. andersonii* orthologs of these genes (Supplementary Figures 11, 12) and compared their expression levels in young root segments of three *Pannsp1, Pannsp2* and control plants by qRT-PCR. This showed that expression of *PanD27* and *PanMAX1* is reduced in roots of *Pannsp1* and *Pannsp2* mutant lines (**Figure 5**). We noted that *Pannsp1* mutant lines differ in the level of *PanD27* and *PanMAX1* expression. Both genes have an intermediate expression level in *Pannsp1–6* and *Pannsp1–13*, compared to *Pannsp1–39* and *Pannsp2* mutants (**Figure 5**). The three *Pannsp1* mutant lines differ from each other in the type of mutations that were created. *Pannsp1–6* and *Pannsp1–13* contain a 1 bp insertion and 5 bp deletion close to the 5′-end of the coding region, respectively. These mutations are immediately followed by a second in-frame ATG that in WT PanNSP1 encodes a methionine at position 16. In contrast, *Pannsp1–39* contains a large 232 bp deletion that removes this in-frame ATG (see Supplementary Figure 9). Together, this suggests that *Pannsp1–6* and *Pannsp1–13* might represent weak alleles. Overall, these data suggest that regulation of *D27* and *MAX1* expression by NSP1 and NSP2 is conserved between *M. truncatula* and *P. andersonii*.

Taken together, we showed that EIN2, HK4, NSP1, and NSP2 in *P. andersonii* commit non-symbiotic functions in hormonal homeostasis. These functions are in line with what is described for other plant species, suggesting that the generated *P. andersonii* lines represent true mutants.

### Nodulation Phenotypes of *Parasponia Panein2* and *Panhk4* Mutants Differ From Their Legume Counterparts

To determine whether *PanEIN2, PanHK4, PanNSP1*, and *PanNSP2* perform similar functions during nodule formation as their legume orthologs, *P. andersonii* mutant plantlets were inoculated with *Mesorhizobium plurifarium* BOR2 (van Velzen et al., 2017). Nodulation phenotypes were examined 1 month after inoculation.

**FIGURE 6:**
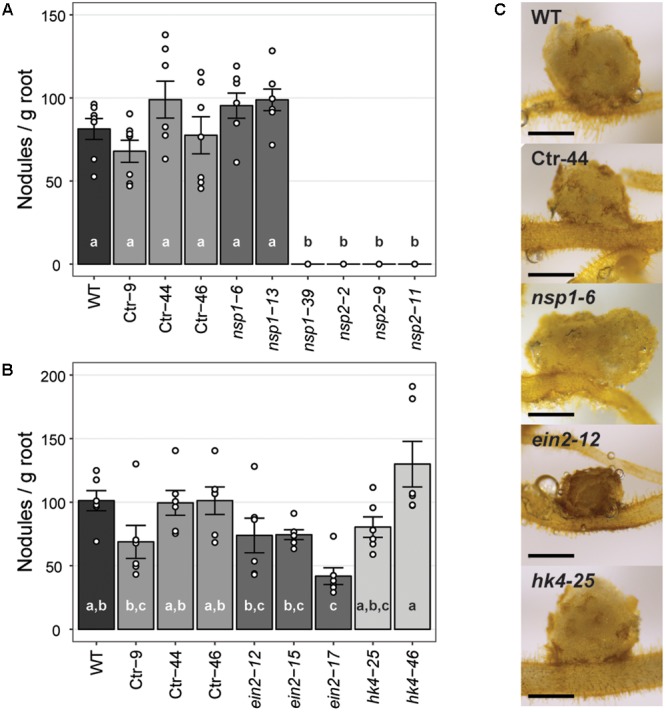
Nodule formation on *P. andersonii* CRISPR/Cas9 mutant lines. **(A)** Nodule number per gram root fresh weight on WT, transgenic control (Ctr) and *Pannsp1* and *Pannsp2* mutant lines. Nodule number was determined at 1 month after inoculation with *Mesorhizobium plurifarium* BOR2. **(B)** Nodule number per gram root fresh weight on WT, transgenic control (Ctr) and *Panein2* and *Panhk4* mutant lines. Nodule number was determined at 1 month after inoculation with *Mesorhizobium plurifarium* BOR2. **(C)** Representative images of 1 month-old nodules. Note the dark color of *Panein2* nodules. Scale bars are equal to 0.5 mm. Data in **(A,B)** represent means of 5–7 biological replicates ± SEM. Dots represent measurement values of biological repeats. Different letters indicate statistical significance (*p* < 0.05) as determined by ANOVA in combination with Tukey *post hoc* test. Data on nodule number and root weight are shown in Supplementary Figure 13.

The strong *Pannsp1–39* mutant allele and all three *Pannsp2* mutant lines are unable to form root nodules (**Figure 6A** and Supplementary Figure 13). This is similar as described for *M. truncatula, L. japonicus*, and *Pisum sativum nsp1* and *nsp2* mutants (Kaló et al., 2005; Smit et al., 2005; Heckmann et al., 2006; Shtark et al., 2016). In contrast, the weak *Pannsp1* alleles *Pannsp1–6* and *Pannsp1–13* could be nodulated similar as WT or control transgenic plants (**Figure 6A** and Supplementary Figure 13), suggesting that residual PanNSP1 activity is sufficient to support root nodule formation. Overall, these data show that NSP1 and NSP2 functioning is essential for root nodule formation in *Parasponia*.

Analysis of the nodulation phenotype of *P. andersonii Panhk4* mutants showed that PanHK4 is not required for root nodule formation. Both *Panhk4* mutant lines formed a similar amount of nodules as WT and transgenic controls (**Figure 6B** and Supplementary Figure 13). This is different from the corresponding legume mutants – *M. truncatula Mtcre1* and *L. japonicus Ljlhk1* – that are generally not forming root nodules (Murray et al., 2007; Plet et al., 2011).

The phenotype of *P. andersonii Panein2* mutants also differs from that of legume mutants. *M. truncatula ein2* mutants – as well as *L. japonicus* plants in which both EIN2-encoding genes have been silenced – form more nodules than WT, which are clustered in distinct zones along the root (Penmetsa and Cook, 1997; Miyata et al., 2013). *Panein2* mutants do not form such nodule clusters and nodule number is not higher than WT (**Figure 6B** and Supplementary Figure 13). However, nodules formed on *Panein2* mutant plants are smaller and dark colored when compared to nodules of control plants (**Figure 6C**). This suggests impaired nodule development in *P. andersonii ein2* mutants.

**FIGURE 7:**
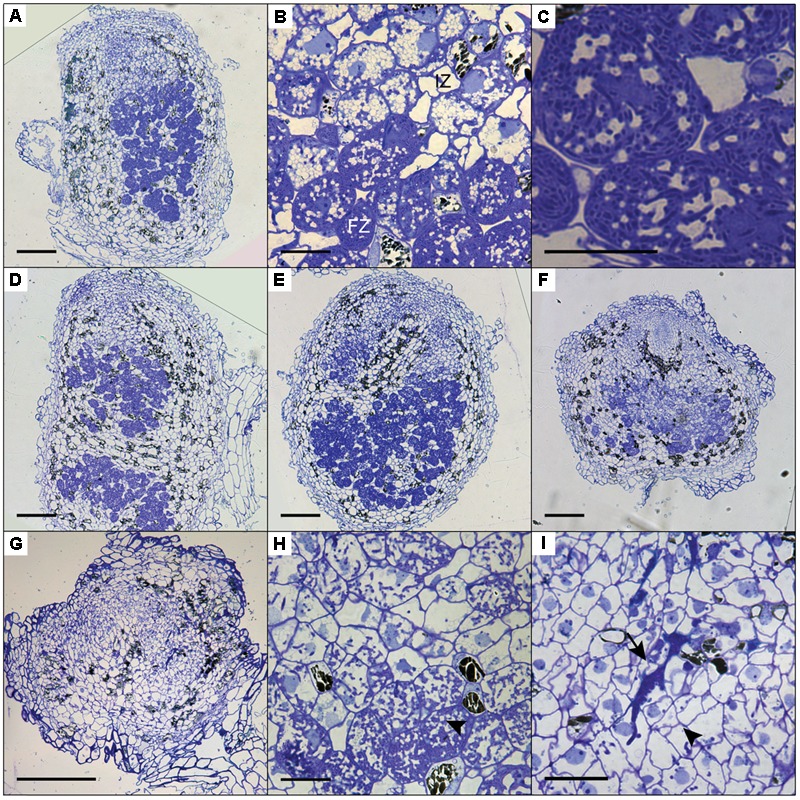
Cytoarchitecture of CRISPR/Cas9 mutant nodules. **(A)** Longitudinal nodule sections of 1 month-old nodule formed on transgenic control line Ctr-9. **(B)** Zoom in on cells in the infection (IZ) and fixation zone (FZ) of the transgenic control nodule shown in **(A)**. Note the presence of small fragmented vacuoles in infected cells in the infection zone. **(C)** Zoom in on a cell in the fixation zone of a transgenic control nodule showing the presence of fixation threads. **(D)** Longitudinal nodule sections of 1 month-old nodule formed on *Pannsp1–13*. **(E)** Longitudinal nodule sections of 1 month-old nodule formed on *Panhk4–25*. **(F)** Longitudinal nodule sections of 1 month-old nodule formed on *Panein2–15*. **(G)** Longitudinal nodule sections of 1 month-old nodule formed on *Panein2–17*. **(H)** Zoom in on cells in the basal part of the *Panein2–15* nodule shown in **(F)**. Indicated by an arrowhead are cells containing fixation threads. **(I)** Zoom in of the *Panein2–17* nodule shown in **(G)**. Indicated by an arrowhead are infection threads. Indicated by an arrow are large apoplastic colonies. Scale bars in **(A,D–G)** are equal to 150 and 25 μm in **(B,C,H,I)**.

To determine the cytoarchitecture of *Panein2, Panhk4*, and *Pannsp1–6/Pannsp1–13* mutant nodules, we sectioned ∼10 nodules for each mutant line and studied these by light microscopy. Wild-type *P. andersonii* nodules harbor an apical meristem, followed by several cell layers that contain infection threads (**Figure 7A**) (Op den Camp et al., 2012). Below this infection zone, 2–3 cell layers are present that display vacuolar fragmentation and increase in size compared to non-infected cells (**Figure 7B**). These cells are followed by cells that are filled with fixation threads (**Figures 7B,C**). The general cytoarchitecture of *Panhk4* and *Pannsp1–6/Pannsp1–13* mutant nodules does not differ from that of WT or transgenic control nodules (**Figures 7A,D,E**), suggesting that these are functional. In contrast, in *Panein2* mutant nodules intracellular infection is hampered (**Figures 7F–I**). Most (>75%) *Panein2* mutant nodules harbor only infection threads as well as large apoplastic colonies (**Figure 7I**). Some mutant nodules, harbor cells that contain fixation threads. However, even in the best nodules, fixation thread formation is severely delayed and many cells in the fixation zone still show vacuolar fragmentation (**Figure 7I**). This shows that ethylene signaling is required for efficient fixation thread formation in *P. andersonii* nodules.

Taken together, these data reveal symbiotic mutant phenotypes for *nsp1, nsp2* and *ein2*, whereas no effect on nodule formation was found by knocking out *hk4* in *P. andersonii.* Interestingly, we uncovered a novel role for the ethylene signaling component EIN2 in intracellular infection of *P. andersonii* nodules.

## Discussion

Comparative studies between legumes and the Cannabaceae tree *Parasponia* can provide insights into ‘core’ genetic networks underlying rhizobium symbiosis (van Velzen et al., 2017). To facilitate such studies, we aimed to establish a reverse genetics platform for *P. andersonii* based on CRISPR/Cas9 genome editing. We show that using *A. tumefaciens* transformation, *P. andersonii* stable transgenic lines can be obtained in 3–4 months. In combination with CRISPR/Cas9 mutagenesis, this allows efficient generation of bi-allelic knockout mutants. As a proof-of-concept, we mutated four genes that commit essential symbiotic functions in legumes as well as control different hormonal networks. Characterization of the resulting lines revealed both symbiotic as well as non-symbiotic mutant phenotypes. Therefore, we conclude that stable *A. tumefaciens*-mediated transformation in combination with CRISPR/Cas9 genome editing can be efficiently used for reverse genetic analysis in *P. andersonii*.

Plant transformation efficiency is the main bottleneck in plant genome editing (Altpeter et al., 2016; Ledford, 2016). Especially regeneration of an entire transgenic plant out of a single transformed cell remains difficult for most plant species. We took advantage of an efficient micro-propagation system available for *Parasponia* spp. to establish a protocol for stable transformation (Davey et al., 1993; Webster et al., 1995; Cao et al., 2012). About 8–12 weeks after cocultivation with *A. tumefaciens*, ∼50% of explants develop transgenic shoots. This relatively high efficiency is, in part, obtained through an initial 9-day culturing period on root-inducing medium, before incubation on standard propagation medium. This adaptation in the protocol was inspired by a recent study that showed that regeneration of plant cells consists of two distinctive steps (Kareem et al., 2015). Regenerative competence is established through activation of a root developmental program, followed by activation of shoot promoting factors that are required to complete shoot regeneration (Kareem et al., 2015). The latter explains why transfer to propagation medium is required to regenerate *P. andersonii* transgenic shoots. However, this promoting effect of rooting medium on regeneration of transgenic shoots might differ between different explant types, as noted for *P. andersonii* stems and petioles (Supplementary Table 3).

An advantage of the *Parasponia* system is that T_0_ transgenic knockout mutants can be clonally propagated through *in vitro* micro-propagation (Davey et al., 1993; Webster et al., 1995; Cao et al., 2012). This allows a large number of rooted plantlets to be generated in a relatively short time span. As a result, phenotypic characterization can be initiated already at 4 months after the start of the transformation. However, a disadvantage of clonal propagation in combination with CRISPR/Cas9 mutagenesis is the possibility of obtaining chimeric mutants. Among the mutant lines we created, we identified one line (out of 11) that was chimeric for one out of three CRISPR target sites (Supplementary Figure 10C). Most mutant lines were genetically homogeneous, suggesting that mutations are induced soon after T-DNA integration. This is consistent with results in poplar, which also revealed a low percentage of chimeric mutants (Fan et al., 2015). Since chimeras are observed occasionally, thorough genotypic analysis will be required when phenotyping is performed in the T_0_ generation. Besides vegetative propagation, *Parasponia* trees can also be propagated generatively. Under suitable greenhouse conditions, *Parasponia* trees flower within ∼6–9 months and are self-compatible (Becking, 1992). However, *Parasponia* trees can be monoecious or diecious and female flowers are wind pollinated (Soepadmo, 1974). This complicates selfing of trees and the production of pure seed badges. Additionally, *Parasponia* trees are fast growing and occupy a substantial amount of space in a tropical greenhouse (28°C, ∼100% relative humidity), making generative propagation of multiple mutant lines logistically somewhat challenging. An alternative to generative propagation is *in vitro* maintenance of transgenic lines. Additionally, the fast and efficient transformation procedure presented here will allow recreation of a particular mutant in less than 6 months.

Among the mutants we created, *Panhk4* and *Panein2* showed symbiotic phenotypes that differ from corresponding legume mutants. *P. andersonii Panhk4* mutants form nodules with a WT cytoarchitecture, indicating that these nodules are most likely functional. Analysis of stem cross-sections showed that *Panhk4* mutants possess a reduced procambial activity. Similar phenotypes are observed in homologous mutants in *A. thaliana* (Mahonen et al., 2006a,b). Procambium activity is slightly reduced in the orthologs receptor mutant *arabidopsis histidine kinase 4* (*ahk4*), whereas it is completely abolished in the *ahk2 ahk3 ahk4* triple mutant (Mahonen et al., 2006a,b). The comparable phenotypes in cambium activity upon mutating histidine kinases suggest that *PanHK4* encodes a functional cytokinin receptor. *M. truncatula* and *L. japonicus* mutants in the cytokinin receptors orthologs to *PanHK4* are characterized as nodulation deficient (Murray et al., 2007; Plet et al., 2011). However, these mutants occasionally form nodules (Plet et al., 2011; Held et al., 2014; Boivin et al., 2016). This suggests redundant functioning of additional cytokinin receptors in both legume species. The *P. andersonii* genome also encodes two additional cytokinin receptors: *PanHK2* and *PanHK3* (van Velzen et al., 2017) (Supplementary Figure 4). Therefore, redundant functioning of one of these receptors cannot be excluded. In legumes, cell divisions associated with nodule development are initiated in the root cortex in response to epidermal perception of rhizobial signals (Timmers et al., 1999; Xiao et al., 2014). Cytokinin appears important for activation of this cortical organogenesis program (Vernie et al., 2015; Gamas et al., 2017). In *Parasponia*, cell divisions associated with nodule development are first observed in the epidermis, the cell layer that is in direct contact with the rhizobium bacteria (Lancelle and Torrey, 1984; Geurts et al., 2016). This difference in mitotically-responding tissues could create different dependencies on cytokinin signaling between legumes and *Parasponia*. However, whether this explains the absence of a symbiotic phenotype of *Panhk4* mutants requires further experimentation.

*Panein2* mutants are ethylene insensitive, as indicated by the absence of leaf abscission following ethylene treatment. Additionally, we noticed a disturbed sex differentiation in *Panein2* flowers. Functioning of ethylene in flower sex differentiation is known in cucurbit species, like cucumber (*Cucumis sativus*) and melon (*Cucumis melo*) (Rudich et al., 1972; Yin and Quinn, 1995; Tanurdzic and Banks, 2004). Molecular genetic studies revealed that flower bud-specific expression of ACC synthase (ACS) genes, which are essential for biosynthesis of the ethylene precursor ACC, inhibits stamen development (Boualem et al., 2008, 2015). In line with these findings in cucurbits, we hypothesize that EIN2-mediated ethylene signaling commits a similar function in sex differentiation in *Parasponia* species.

In symbiotic context, *EIN2* knockout mutations result in different phenotypes between *Parasponia* and legumes. In legumes, ethylene negatively regulates rhizobial infection and root nodule formation (Penmetsa and Cook, 1997; Penmetsa et al., 2008; Miyata et al., 2013). This is illustrated by the phenotype of the *M. truncatula ein2* mutant (named *sickle*) that forms extensive epidermal infection threads and clusters of small nodules (Penmetsa and Cook, 1997; Xiao et al., 2014). *P. andersonii ein2* mutants also form smaller nodules than WT. However, in contrast to the *Mtein2* mutant, these nodules are regularly spaced on the root system. This suggests that in *Parasponia* ethylene signaling is not involved in regulating nodule number. Additionally, also the infection phenotype of *Panein2* mutants differs from that in legumes. In *M. truncatula* and *L. japonicus*, interference with ethylene signaling increases the number of epidermal infection threads but does not affect intracellular colonization of nodule cells (Penmetsa and Cook, 1997; Nukui et al., 2004; Lohar et al., 2009). In contrast, in *P. andersonii Panein2* mutants, intracellular colonization is hampered. Inside nodules, large apoplastic colonies are observed and fixation thread formation is severely reduced or even absent. This suggests that in *Parasponia* a functional ethylene signaling pathway is required for efficient intracellular infection of nodule cells.

Mutagenesis of the *NSP2* ortholog of *P. andersonii* indicated a conserved symbiotic role for this GRAS-type transcriptional regulator. In legumes, NSP2 works in concert with NSP1 to control root nodule formation (Hirsch et al., 2009). Mutagenesis of the *NSP1* ortholog of *P. andersonii* resulted in contrasting nodulation phenotypes. Two mutant lines, *Pannsp1–6* and *Pannsp1–13*, form nodules with a WT cytoarchitecture, whereas mutant line *Pannsp1–39* is unable to form nodules (**Figures 6, 7**). However, all three mutants are affected in transcriptional regulation of strigolactone biosynthesis genes *PanD27* and *PanMAX1* (**Figure 5**). The three *Pannsp1* mutant lines differ from each other in the type of mutations that were created. *Pannsp1–6* and *Pannsp1–13* contain small deletions that are immediately followed by a second in-frame ATG that in WT PanNSP1 encodes a methionine at position 16. In contrast, *Pannsp1–39* contains a larger deletion that removes this in-frame ATG (see Supplementary Figure 9). Several reports have shown that alternative start codons are occasionally used to initiate transcription (Chabregas et al., 2003; Thatcher et al., 2007; Bazykin and Kochetov, 2011). Therefore, *Pannsp1–6* and *Pannsp1–13* most probably represent weak alleles that still possess residual PanNSP1 function. Such residual levels of PanNSP1 are affecting the expression of strigolactone biosynthesis genes, but are still sufficient to allow nodule formation. Therefore, we argue that the *P. andersonii Pannsp1–39* line carries a knockout mutation, indicating that in *P. andersonii* both NSP1 and NSP2 are essential for rhizobium root nodule formation.

Taken together, we showed that *P. andersonii* can be efficiently transformed using *A. tumefaciens* and is amenable to targeted mutagenesis using CRISPR/Cas9. This protocol takes only marginally more time than the transient *A. rhizogenes* transformation system that is generally used to study root nodule formation (e.g., Boisson-Dernier et al., 2001; Kumagai and Kouchi, 2003; Limpens et al., 2004; Op den Camp et al., 2011; Cao et al., 2012) but has several advantages. One of these is the absence of the *A. rhizogenes root inducing locus* (*rol*) that interferes with hormone homeostasis (Nilsson and Olsson, 1997). The protocol we developed will allow studies on *P. andersonii* symbiosis genes to determine to what extent legumes and *Parasponia* use a similar mechanism to establish a nitrogen-fixing symbiosis with rhizobium.

## Data Availability Statement

All datasets analyzed for this study are included in the manuscript and the supplementary files. Gene identifiers for all *P. andersonii* genes used in this study can be found in Supplementary Table 8. Sequences can be downloaded from www.parasponia.org.

## Author Contributions

Conceptualization, AvZ and RG; Methodology, AvZ, MH, SL, and WK; Investigation, AvZ, TW, MSK, LR, FB, MH, SL, EF, and WK; Formal analysis, AvZ, TW, and EF; Visualization, AvZ; Writing – original draft, AvZ; Writing – review and editing, AvZ and RG; Funding acquisition, TB and RG; Supervision, RG.

## Conflict of Interest Statement

The authors declare that the research was conducted in the absence of any commercial or financial relationships that could be construed as a potential conflict of interest.

## Footnotes

1 http://phytozome.jgi.doe.gov/

2 www.arabidopsis.org

3 http://tree.bio.ed.ac.uk/software/figtree/

4 www.arabidopsis.com
