# Supplementary material for: CRISPR/Cas9-Mediated Mutagenesis of Four Putative Symbiosis Genes of the Tropical Tree Parasponia andersonii Reveals Novel Phenotypes
Source: Front Plant Sci. 2018 Mar 6;9:284. doi: 10.3389/fpls.2018.00284 (PMC5845686; doi:10.3389/fpls.2018.00284)
Supplement: Supplementary file 1 [file Data_Sheet_1.PDF]

SUPPLEMENTAL INFORMATION

The data presented in this document belong to the manuscript entitled:

CRISPR/Cas9-Mediated Mutagenesis of Four Putative Symbiosis Genes of the Tropical Tree *Parasponia andersonii* Reveals Novel Phenotypes

Arjan van Zeijl, Titis A. K. Wardhani, Maryam Seifi-Kalhor, Luuk Rutten, Fengjiao Bu, Marijke Hartog, Sidney Linders, Elena Fedorova, Ton Bisseling, Wouter Kohlen and Rene Geurts

Supplemental Table 1. List of Golden Gate[a] constructs used in this study.

Construct	Description	Level	Backbone	Contains[b]	Literature
1	*nptII* resistance cassette	1	pICH47802	pICSL70004:*nptII*	-
2	$35S_{pro}$:ΩNLS-Cas9:$35S_{ter}$	1	pICH47742	pICH41388:$35S_{pro}$, pAGM5331:ΩNLS, pICH41308::aCas9, pICH41414:$35S_{ter}$	Fauser et al. (2014)
3	PanEIN2sgRNA1	1	pICH47751	pICSL01009:AtU6p, corresponding PCR amplicon	Nekrasov et al. (2013)
4	PanNSP1sgRNA1	1	pICH47751	pICSL01009:AtU6p, corresponding PCR amplicon	Nekrasov et al. (2013)
5	PanNSP2sgRNA1	1	pICH47751	pICSL01009:AtU6p, corresponding PCR amplicon	Nekrasov et al. (2013)
6	PanNSP2sgRNA2	1	pICH47761	pICSL01009:AtU6p, corresponding PCR amplicon	Nekrasov et al. (2013)
7	PanNSP2sgRNA3	1	pICH47841	pICSL01009:AtU6p, corresponding PCR amplicon	Nekrasov et al. (2013)
8	CRISPR_ctrl	2	pICSL4723	1R: construct 1, 2F: construct 2, end-link pICH41744	-
9	CRISPR_PanEIN2	2	pICSL4723	1R: construct 1, 2F: construct 2, 3F: construct 3, end-link pICH41766	-
10	CRISPR_PanNSP1	2	pICSL4723	1R: construct 1, 2F: construct 2, 3F: construct 4, end-link pICH41766	-
11	CRISPR_PanNSP2	2	pICSL4723	1R: construct 1, 2F: construct 2, 3F: construct 5, 4F: construct 6, 5R: construct 7, end-link pICH41800	-

[a] Backbones and standard parts used for Golden Gate assembly are from Engler et al. (2014).
[b] 1F, 2R, etc. indicate position and orientation in level 2 constructs. Construct followed by a number refers to constructs build during this study and described in this table.

Supplemental Table 2. Primers used in this study.

Name	Purpose	Sequence
PanEIN2_sgRNA1	Clone sgRNA	tgtggtctcaattGTGTTAATCCTGGA AAATGGGgttttagagctagaaatagcaag
PanNSP1_sgRNA1	Clone sgRNA	tgtggtctcaattGCCAGTCCATGATG TGATCCGgttttagagctagaaatagcaag
PanNSP2_sgRNA1	Clone sgRNA	tgtggtctcaattGAAGACGACCATGG CTGCGCGgttttagagctagaaatagcaag
PanNSP2_sgRNA2	Clone sgRNA	tgtggtctcaattGACTGGAACGTCC TTACCGGgttttagagctagaaatagcaag
PanNSP2_sgRNA3	Clone sgRNA	tgtggtctcaattGTAGTTCGACACCG CCTACGGgttttagagctagaaatagcaag
sgRNA_Rv	Clone sgRNA	tgtggtctcaAGCGTAATGCCAACTTTGTAC
geno_Cas9_Fw	Amplify aCas9	TTCGATCTCGCTGAGGATGC
geno_Cas9_Rv	Amplify aCas9	TAGCGAGAGGTCCCACGTAG
geno_PanEIN2-crispr-Fw	Genotyping CRISPR mutants	CATTGCAACAGCCTGTGGAC
geno_PanEIN2-crispr-Rv	Genotyping CRISPR mutants	CTGAGCAAGACCCCTTCCAG
geno_PanHK4-crispr-Fw	Genotyping CRISPR mutants	ATGCAGAACCACCATTCCGT
geno_PanHK4-crispr-Rv	Genotyping CRISPR mutants	GCATACTCATCCCGGACAGG
geno_PanNSP1-crispr-Fw	Genotyping CRISPR mutants	TGGTTTCTCGTGGCCTTTGT
geno_PanNSP1-crispr-Rv	Genotyping CRISPR mutants	CTGTGGCCTTAGCTGAGCTT
geno_PanNSP2-crispr-Fw	Genotyping CRISPR mutants	ACTTCCACTCTGTCCCCGAA
geno_PanNSP2-crispr-Rv	Genotyping CRISPR mutants	GACCGGTGACTGAAGTGAGG
qPanD27_Fw	qRT-PCR	TCGGATCGCCATTCAACATC
qPanD27_Rv	qRT-PCR	GACAAGTTCCCGCTGTTTTG
qPanMAX1_Fw	qRT-PCR	TGATGAGATCGTGGCCAAGAG
qPanMAX1_Rv	qRT-PCR	AAAAACGTTCCTCGCAACCG
qPanUNK2_Fw	qRT-PCR	TGCCATTGGTGTTAGCTGTG
qPanUNK2_Rv	qRT-PCR	GTGTCTATCACTGCCTCTTTGC
qPanEF1α_Fw	qRT-PCR	AGACAAGGTTAAGCGTGCAG
qPanEF1α_Rv	qRT-PCR	TGCAACTGGGCAACAAACTC
qTCN-1_Fw	T-DNA copy number	AAGCGCGTTACAAGAAAGCC
qTCN-1_Rv	T-DNA copy number	ACGTTGCCCGCATAATTACG
qTCN-3_Fw	T-DNA copy number	TGCACGACCACGCATTAATG
qTCN-3_Rv	T-DNA copy number	ACGATGCCATGTTCATCTGC
PanAGT1_Fw	T-DNA copy number	AGCGCTTGATCTCCTTTTCG
PanAGT1_Rv	T-DNA copy number	TGCTTTACCTTGTGGCAGTC
Pan338920_Fw	T-DNA copy number	TGAAGCACAACACACGATGG
Pan338920_Rv	T-DNA copy number	TGCTTTCAGCCAGTTAACCG
nptII_Fw	Southern blotting	CACAACAGACAATCGGCTGC
nptII_Rv	Southern blotting	TGATATTCGGCAAGCAGGCA

Supplemental Table 3. Composition of tissue culture media.

Component	Medium		
	SH10	Propagation	Root-inducing
Schenk & Hildebrandt basal salt medium[a,b]	1x	1x	1x
Schenk & Hildebrandt vitamin mixture[a,b]	1x	1x	1x
Sucrose[a]	1% (w/v)	1% (w/v)	2% (w/v)
BAP (6-Benzylaminopurine)[c]	-	1 mg/l	-
IBA (Indole-3-butyric acid)[c]	-	0.1 mg/l	1 mg/l
NAA (1-Naphthaleneacetic acid)[c]	-	-	0.1 mg/l
MES (2-ethanesulfonic acid)[c]	3 mM, pH = 5.8	3 mM, pH = 5.8	3 mM, pH = 5.8
Daishin agar[a]	-	0.8% (w/v)	0.8% (w/v)

[a] Duchefa Biochemie, Haarlem, The Netherlands
[b] Schenk and Hildebrandt, 1972
[c] Sigma, St. Louis, USA

Supplemental Table 4. Regeneration efficiency of different explant types. Regeneration was determined for non-transgenic material incubated on propagation medium.

Explant type	Regeneration efficiency
Young leaves	-
Mature leaves	+/-
Midveins	+/-
Stem pieces (5 mm)	++
Stem pieces (1 mm)	+/-
Stem pieces, cut length-wise	+
Petioles	+++
Shoot apical meristems	-
Tissue culture shoots	+

+++, means that > 90% of explants develop multiple regenerative calli.
-, indicates that no regeneration was observed, tissue turned dark brown in color.

Supplemental Table 5. Hormone composition of tissue culture media used in this study.

Hormone	Propagation medium	Root-inducing medium	Alternative media								
			1	2	3	4	5	6	7	8	9
BAP (mg/L)	1	-	0.33	1	3	0.33	1	3	0.33	1	3
IBA (mg/L)	0.1	1	-	-	-	-	-	-	-	-	-
NAA(mg/L)	-	0.1	0.03	0.03	0.03	0.1	0.1	0.1	0.3	0.3	0.3

Supplemental Table 6. Regeneration of transgenic material after co-cultivation and continuous culturing on propagation medium (Prop) or co-cultivation and culturing for seven additional days on root-inducing medium and subsequent transfer to propagation medium (Root -> prop). Regeneration was scored 6 weeks after start of transformation.

	Prop	Root -> prop
Petioles	+	++
Stem pieces (5 mm)	+/-	++

++, means that > 50% of explants develop regenerative calli.
-, means that < 20% of explants develop regenerative calli.

Supplemental Table 7. Molecular characterization of transgenic lines.

Line[a]	T-DNA copy number estimate		Ploidy	Presence of right border sequences[d]
	qRT-PCR[b]	Southern blot[c]		
1	2.9	na	Diploid	+
3*	1.0	1	Diploid	+
4	1.2	na	Diploid	+
12	0.9	na	Diploid	+
13	1.0	2	Diploid	+
18	1.9	na	Diploid	+
19	2.9	3	Diploid	+
21	1.1	na	Diploid	+
31	1.9	2	Diploid	+
33*	1.0	1	Diploid	+
37	2.1	na	Diploid	+
43*	0.9	1	Diploid	+
47	2.8	na	Diploid	+
49	1.1	na	Diploid	+
50	nd	na	Diploid	-
52	1.0	1	Diploid	+
54	0.5	2	Diploid	+
55	0.8	na	Diploid	+
59	0.4	na	Diploid	+
61	0.9	na	Diploid	+

[a] Asterisks indicate lines selected for further analyses.
[b] These numbers indicate measurement values from qRT-PCR on genomic DNA. nd indicates that T-DNA was not detected.
[c] na indicates samples not analyzed by Southern blot.
[d] PCR was performed using primers amplifying a sequence close to the T-DNA right border. +/- indicates presence/absence of a PCR amplicon.

Supplemental Table 8. GeneIDs for all *P. andersonii* genes used in this study. GeneIDs refer to *P. andersonii* gene models, which can be searched for on www.parasponia.org.

Name	GeneID
PanEIN2	PanWU01x14_asm01_ann01_090380
PanHK2	PanWU01x14_asm01_ann01_002510
PanHK3	PanWU01x14_asm01_ann01_222240
PanHK4	PanWU01x14_asm01_ann01_103390
PanNSP1	PanWU01x14_asm01_ann01_334190
PanSCL26	PanWU01x14_asm01_ann01_236490
PanSCL34	PanWU01x14_asm01_ann01_107700
PanSCL16	PanWU01x14_asm01_ann01_130270
PanSHR	PanWU01x14_asm01_ann01_108080
PanNSP2	PanWU01x14_asm01_ann01_157260
PanSCL28	PanWU01x14_asm01_ann01_101430
PanSCL18	PanWU01x14_asm01_ann01_295370
PanSCL17	PanWU01x14_asm01_ann01_245480
PanSCL27	PanWU01x14_asm01_ann01_245490
PanRAM1	PanWU01x14_asm01_ann01_146390
PanD27	PanWU01x14_asm01_ann01_194930
PanD27L1	PanWU01x14_asm01_ann01_057040
PanD27L2	PanWU01x14_asm01_ann01_143920
PanMAX1	PanWU01x14_asm01_ann01_182160
PanUNK2	PanWU01x14_asm01_ann01_211960
PanEF1α	PanWU01x14_asm01_ann01_070680
PanAGT1	PanWU01x14_asm01_ann01_040540
-	PanWU01x14_asm01_ann01_338920

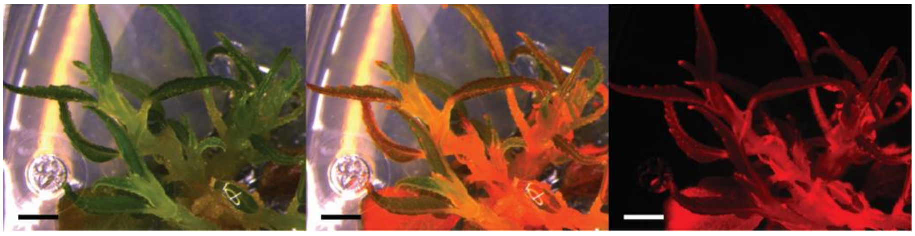

Supplemental Figure 1. *P. andersonii* transgenic shoots can be effectively propagated.
Transgenic *P. andersonii* shoots propagated *in vitro*. Images were taken six months after transformation. Scale bars are equal to 2.5 mm. Shown from left to right are bright-field images, overlays of bright-field and DsRED fluorescence and DsRED fluorescence images.

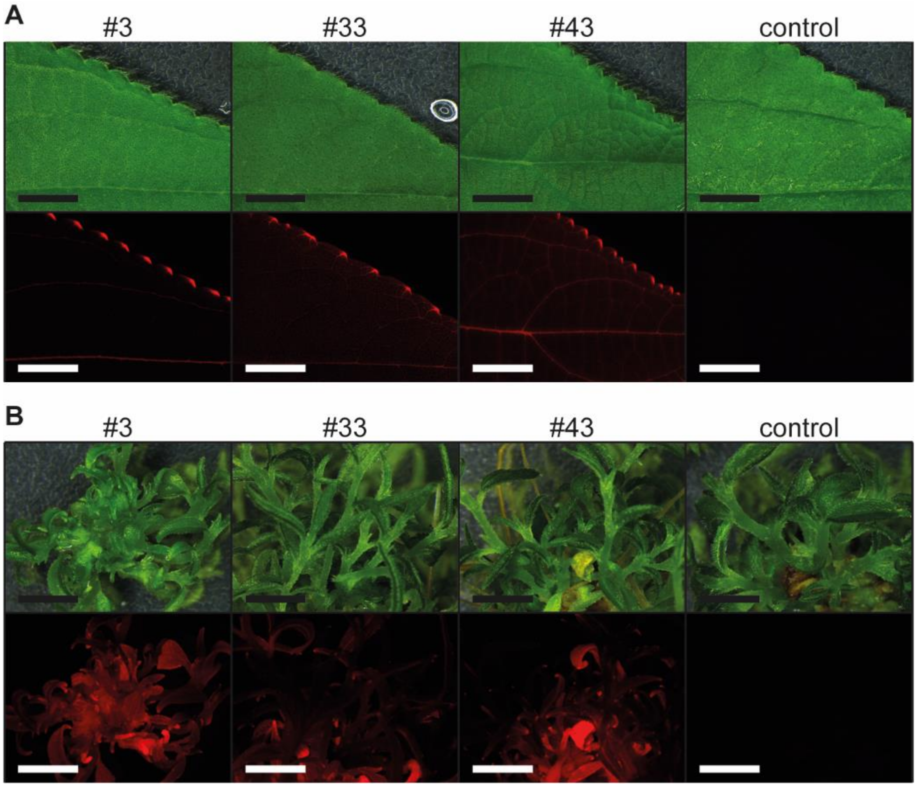

Supplemental Figure 2. *Trans*-genes remain stably integrated in the *P. andersonii* genome.
Bright field (top) and DsRED1 fluorescence (bottom) images of leaves harvested from mature trees grown under greenhouse conditions **(A)** or *in vitro* propagated material **(B)**. Images are shown for transgenic lines 3, 33 and 43 and control plants not expressing the DsRED1 marker. Images were taken one year after transgenic lines were selected. Scale bars are equal to 5 mm.

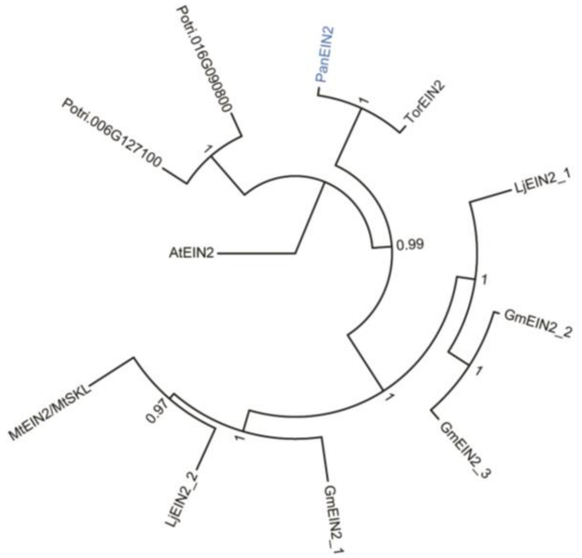

Supplemental Figure 3. Phylogeny of EIN2 proteins.

Phylogeny was reconstructed based on an alignment of EIN2 proteins from *Arabidopsis thaliana* (At), soybean (*Glycine max*, Gm), *Lotus japonicus* (Lj), *Medicago truncatula* (Mt), poplar (*Populus trichocarpa*, Potri), *Parasponia andersonii* (Pan) and *Trema orientalis* (Tor). Branch support is indicated by FastTree support values (Price et al., 2009). Terminals are labeled by their gene name or gene identifier. The EIN2 protein of *P. andersonii* is highlighted in blue. Mid-point rooting was applied for better tree visualization.

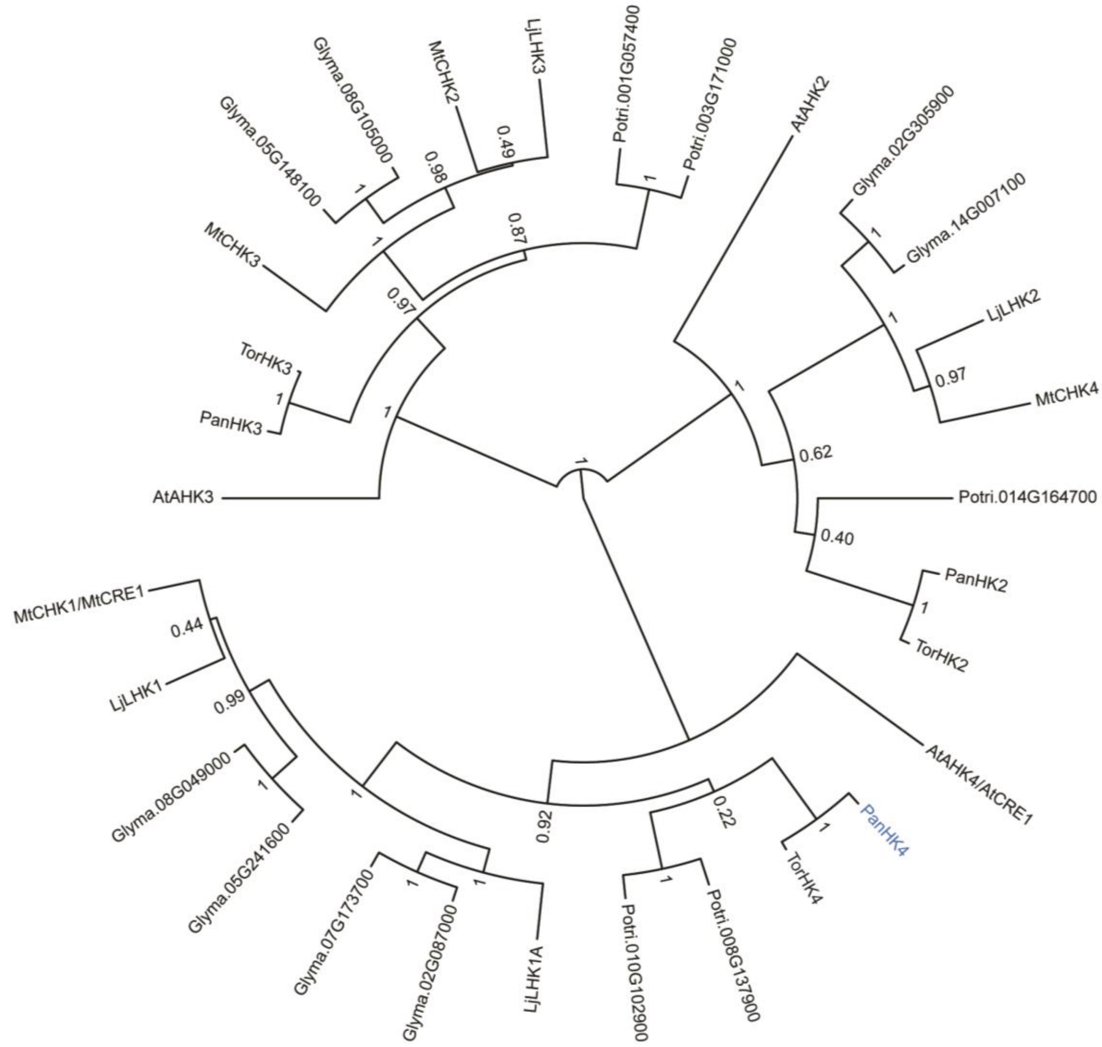

Supplemental Figure 4. Phylogeny of the histidine kinase cytokinin receptor family.
Phylogeny was reconstructed based on an alignment of cytokinin receptor proteins from *Arabidopsis thaliana* (At), soybean (*Glycine max*, Gm), *Lotus japonicus* (Lj), *Medicago truncatula* (Mt), poplar (*Populus trichocarpa*, Potri), *Parasponia andersonii* (Pan) and *Trema orientalis* (Tor). Branch support is indicated by FastTree support values (Price et al., 2009). Terminals are labeled by their gene name or gene identifier. The MtCRE1/LjLHK1 putative orthologue of *P. andersonii* is highlighted in blue. Mid-point rooting was applied for better tree visualization.

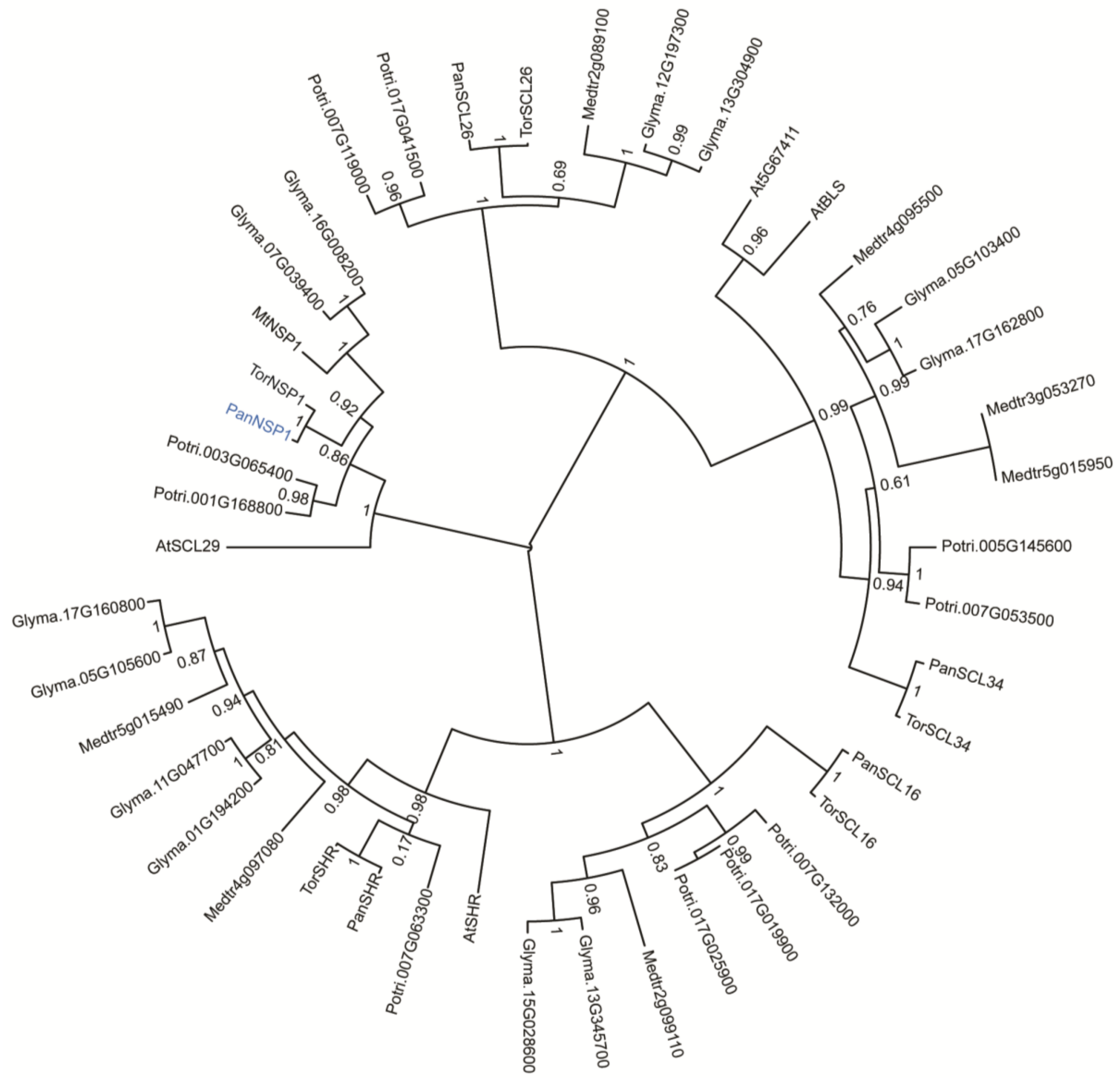

Supplemental Figure 5. Phylogeny of NSP1 and related GRAS proteins.
Phylogeny was reconstructed based on an alignment of GRAS proteins from *Arabidopsis thaliana* (At), soybean (*Glycine max*, Gm), *Medicago truncatula* (Mt), poplar (*Populus trichocarpa*, Potri), *Parasponia andersonii* (Pan) and *Trema orientalis* (Tor). Branch support is indicated by FastTree support values (Price et al., 2009). Terminals are labeled by their gene name or gene identifier. The MtNSP1 putative orthologue of *P. andersonii* is highlighted in blue. Mid-point rooting was applied for better tree visualization.

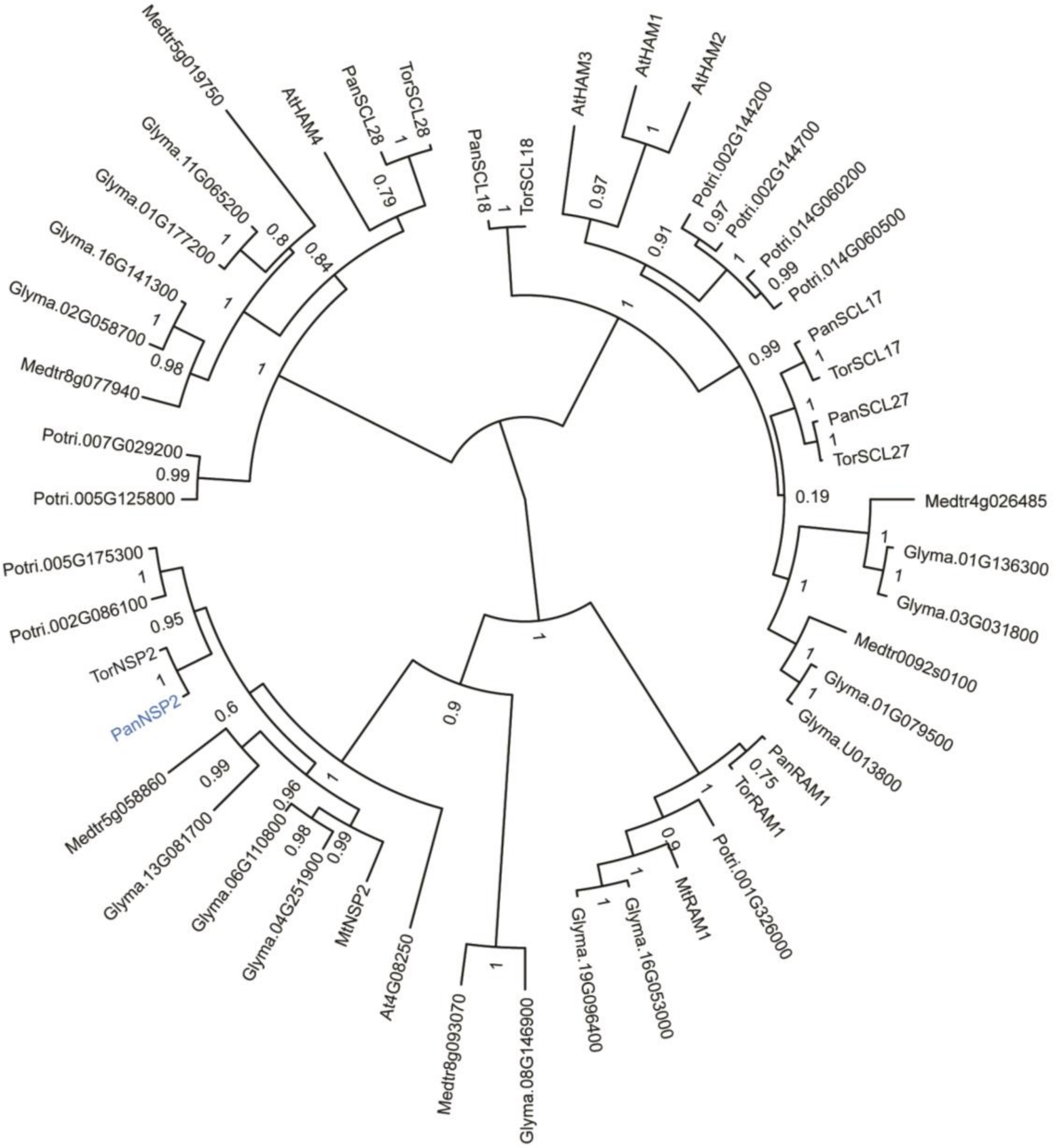

Supplemental Figure 6. Phylogeny of NSP2 and related GRAS proteins.

Phylogeny was reconstructed based on an alignment of GRAS proteins from *Arabidopsis thaliana* (At), soybean (*Glycine max*, Gm), *Medicago truncatula* (Mt), poplar (*Populus trichocarpa*, Potri), *Parasponia andersonii* (Pan) and *Trema orientalis* (Tor). Branch support is indicated by FastTree support values (Price et al., 2009). Terminals are labeled by their gene name or gene identifier. The MtNSP2 putative orthologue of *P. andersonii* is highlighted in blue. Mid-point rooting was applied for better tree visualization.

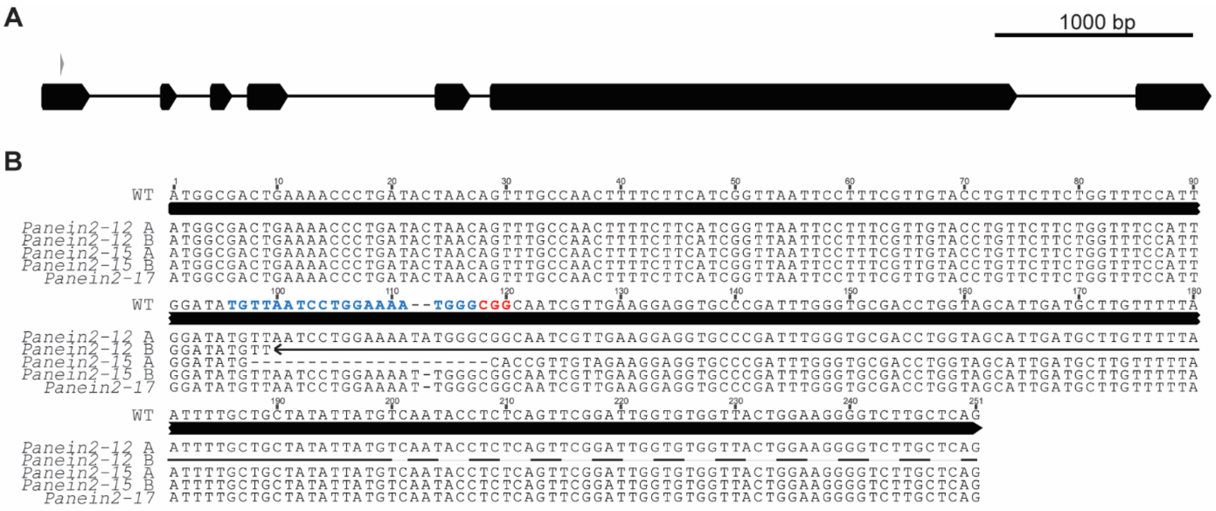

Supplemental Figures 7. CRISPR mutant alleles of *Panein2* mutant lines.

(**A**) Schematic representation of *PanEIN2* gene model. Indicated by a grey arrowhead is the location of the sgRNA target site.

(**B**) Sequence alignment of the first exon of *PanEIN2* in wild type (WT) and *Panein2* mutant lines. For bi-allelic mutant lines, both alleles are shown (A and B alleles). Highlighted in blue and red are the sgRNA target site and PAM sequence, respectively. Arrow in sequence indicates a large (> 100 bp) insertion in *Panein2-12* allele B.

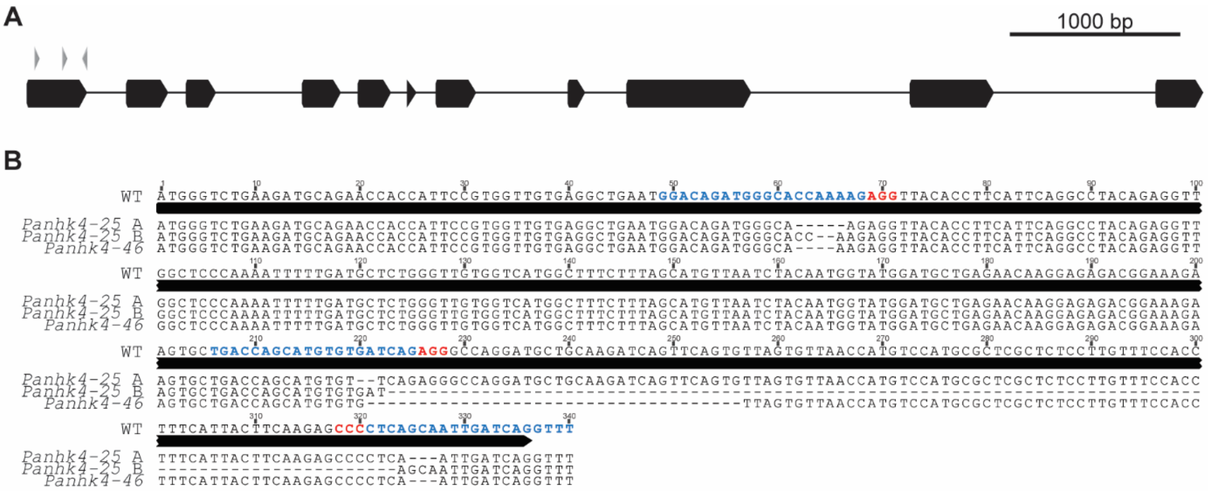

Supplemental Figures 8. CRISPR mutant alleles of *Panhk4* mutant lines.

(**A**) Schematic representation of *PanHK4* gene model. Indicated by grey arrowheads are the locations of three sgRNA target sites.

(**B**) Sequence alignment of the first exon of *PanHK4* in wild type (WT) and *Panhk4* mutant lines. For bi-allelic mutant lines, both alleles are shown (A and B alleles). Highlighted in blue and red are the sgRNA target sites and PAM sequences, respectively.

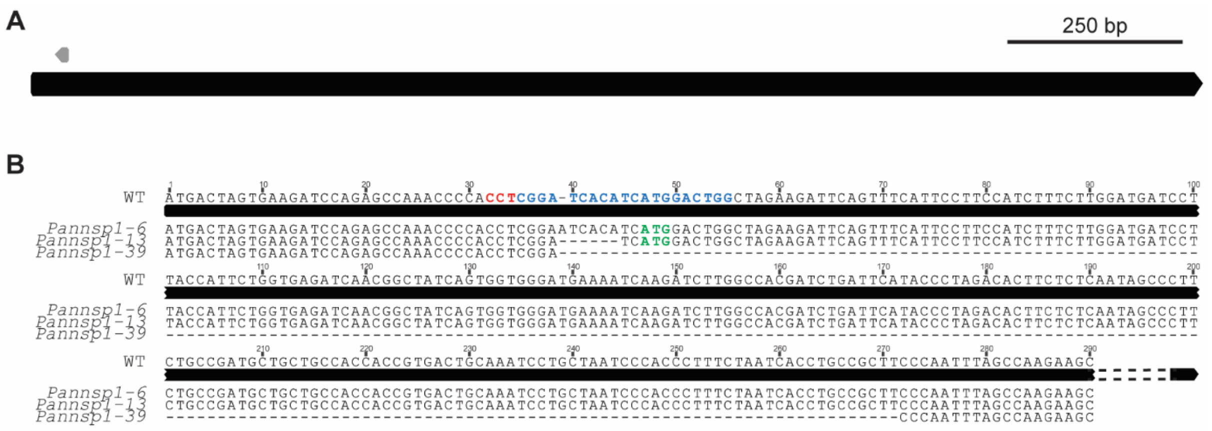

Supplemental Figures 9. CRISPR mutant alleles of *Pannsp1* mutant lines.

(**A**) Schematic representation of *PanNSP1* gene model. Indicated by a grey arrowhead is the locations of the sgRNA target site.

(**B**) Sequence alignment of the first part of *PanNSP1* in wild type (WT) and *Pannsp1* mutant lines. Highlighted in blue and red are the sgRNA target site and PAM sequence, respectively. Highlighted in green are in-frame ATGs present in *Pannsp1-6* and *Pannsp1-13*.

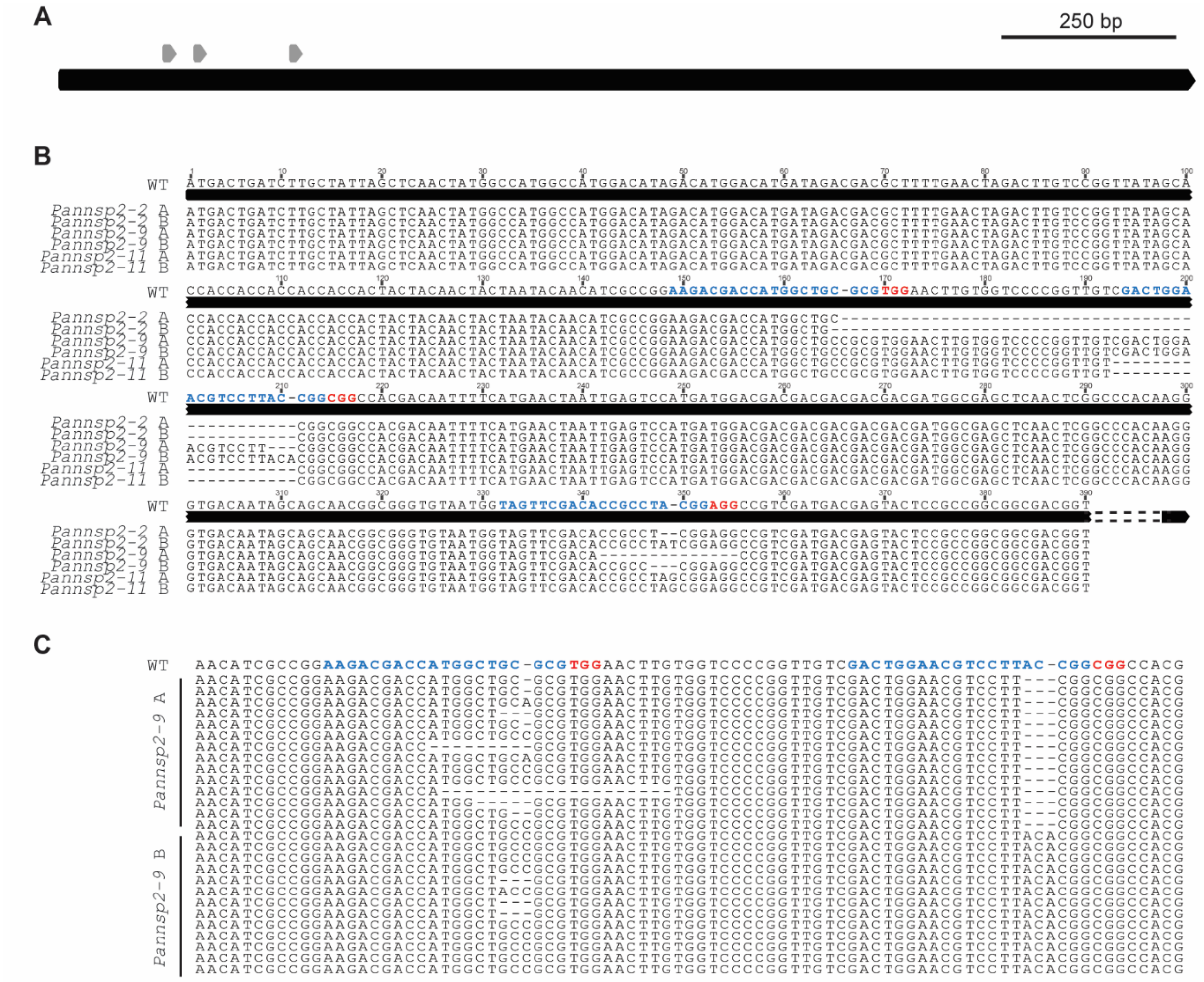

Supplemental Figures 10. CRISPR mutant alleles of *Pannsp2* mutant lines.

(A) Schematic representation of *PanNSP2* gene model. Indicated by grey arrowheads are the locations of three sgRNA target sites.

(B) Sequence alignment of the first part of the *PanNSP2* gene in wild type (WT) and *Pannsp2* mutant lines. For bi-allelic mutant lines, both alleles are shown (A and B alleles). Highlighted in blue and red are the sgRNA target sites and PAM sequences, respectively.

(C) Sequence alignment of part of the *PanNSP2* gene in wild type (WT) and *Pannsp2-9*. The region depicted corresponds to the first two sgRNA target sites. Sequences from multiple individual *Pannsp2-9* shoots are shown, to indicate that this line is chimeric for the first sgRNA target site. Highlighted in blue and red are the sgRNA target sites and PAM sequences, respectively.

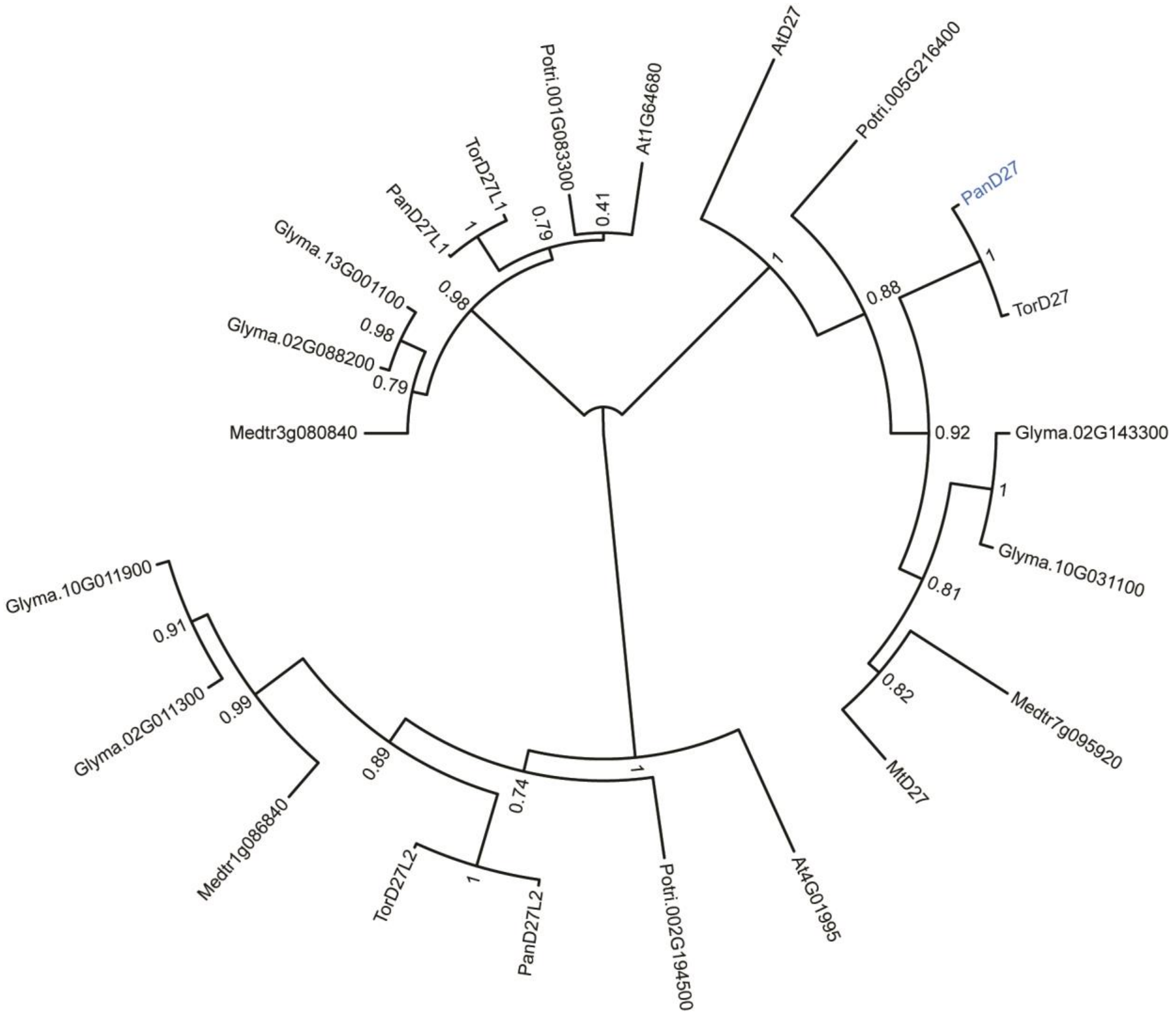

Supplemental Figures 11. Phylogeny of D27 and D27-like proteins.

Phylogeny was reconstructed based on an alignment of D27 and D27-like proteins from *Arabidopsis thaliana* (At), soybean (*Glycine max*, Gm), *Medicago truncatula* (Mt), poplar (*Populus trichocarpa*, Potri), *Parasponia andersonii* (Pan) and *Trema orientalis* (Tor). Branch support is indicated by FastTree support values (Price et al., 2009). Terminals are labeled by their gene name or gene identifier. The *P. andersonii* putative orthologue of MtD27 is highlighted in blue. Mid-point rooting was applied for better tree visualization.

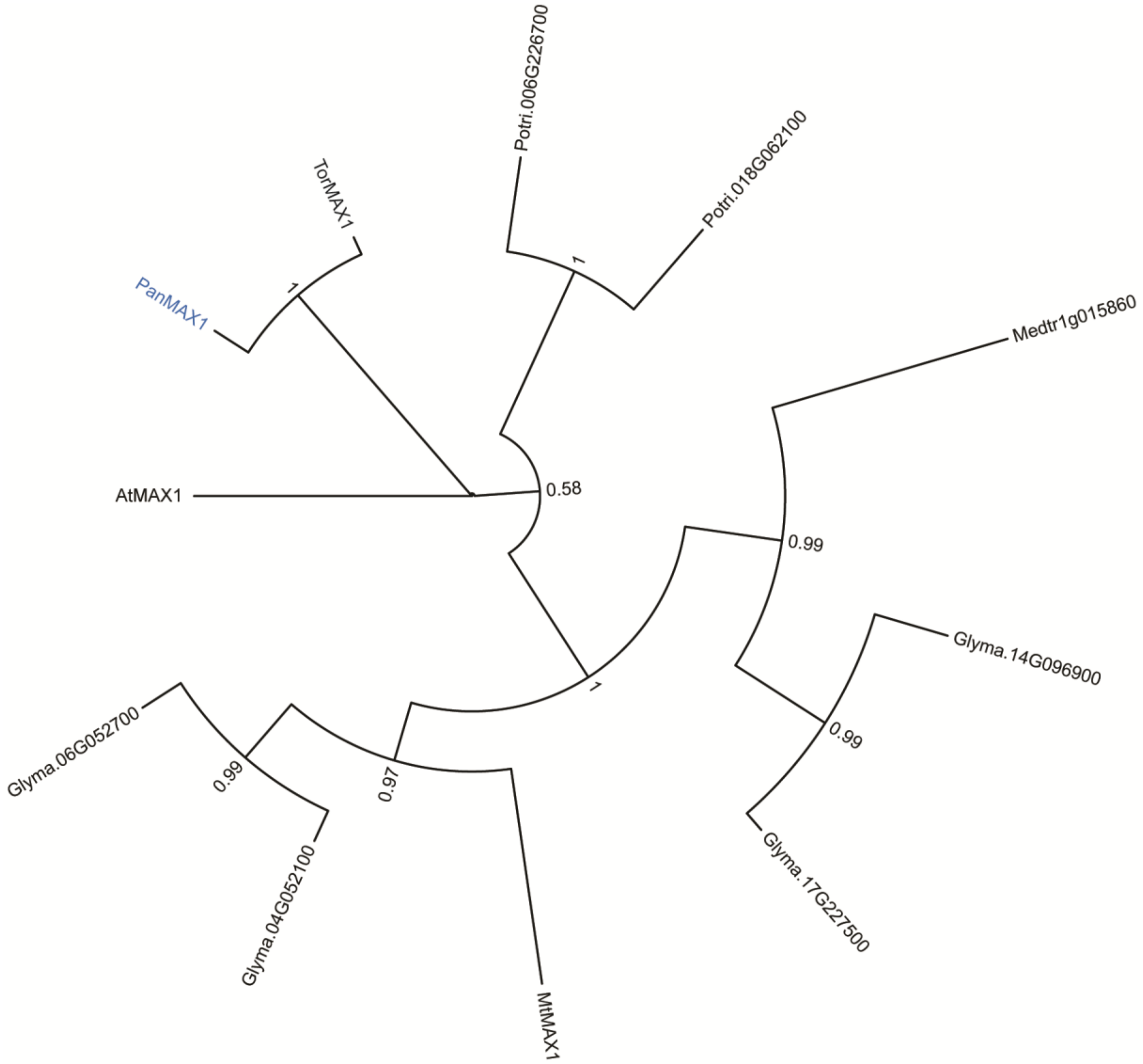

Supplemental Figures 12. Phylogeny of MAX1 proteins.

Phylogeny was reconstructed based on an alignment of MAX1 proteins from *Arabidopsis thaliana* (At), soybean (*Glycine max*, Gm), *Medicago truncatula* (Mt), poplar (*Populus trichocarpa*, Potri), *Parasponia andersonii* (Pan) and *Trema orientalis* (Tor). Branch support is indicated by FastTree support values (Price et al., 2009). Terminals are labeled by their gene name or gene identifier. The *P. andersonii* putative orthologue of MtMAX1 is highlighted in blue. Mid-point rooting was applied for better tree visualization.

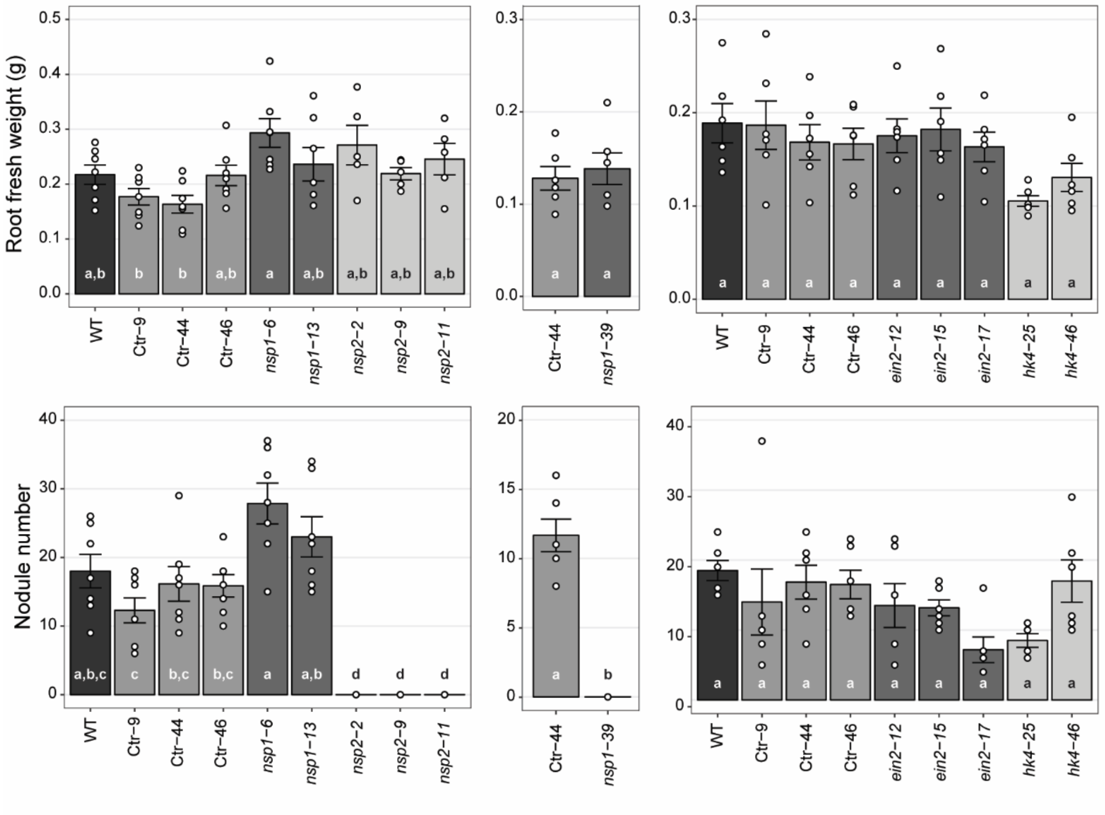

Supplemental Figure 13. Nodule formation on *P. andersonii* CRISPR/Cas9 mutant lines.

Nodule number and fresh root weight data belonging to **Figure 6**. Nodule number and root fresh weight was determined at one month post inoculation with *Mesorhizobium plurifarium* BOR2. Data represent means of 5-7 biological replicates ± SEM. Dots represent measurement values of biological repeats. Different letters indicate statistical significance ($p < 0.05$) as determined by ANOVA in combination with Tukey post-hoc test. WT, wild type.
